# The Flowery Stations Contest in Early Twentieth-Century Italy: Railway Gardens and the Case Study of Ripafratta (Tuscany)

**DOI:** 10.3390/plants15152282

**Published:** 2026-07-25

**Authors:** Athos Pedrelli, Marzia Vergine, Luigi De Bellis, Andrea Luvisi

**Affiliations:** 1 Department of Biological and Environmental Sciences and Technologies, University of Salento, 73100 Lecce, Italy; marzia.vergine@unisalento.it (M.V.); luigi.debellis@unisalento.it (L.D.B.); andrea.luvisi@unisalento.it (A.L.); 2 National Biodiversity Future Center, 90133 Palermo, Italy

**Keywords:** greenery, *Stazioni fiorite*, ornamental plants, railway, renewal, urban area

## Abstract

Urban green areas are essential for human well-being, biodiversity, and environmental sustainability. Although railway renewal projects are celebrated today, Italy pioneered the Flowery Stations Contest (FSC) in the early twentieth century. This study provides the first comprehensive synthesis of the FSC experience (1911–1915), investigating its historical evolution, organizational features, and botanical aspects. A systematic review of 81 historical documents was conducted to reconstruct the contest’s framework. To evaluate long-term impact, we surveyed 90 historically top-ranked stations via satellite imagery and investigated the case study of Ripafratta station (a fraction of San Giuliano Terme, Pisa, Tuscany). Results show the FSC involved up to 291 stations, prioritizing esthetic decorum and hygiene against locomotive soot. Although organizers recommended 64% non-native genera, stationmasters favoured local biodiversity, increasing effectively employed native genera to 43%. Nowadays, 54% of surveyed stations retain their gardens, mainly located in rural areas, yet 82% of these are neglected following the decline of the resident stationmaster’s role. We conclude that the FSC established a significant physical and cultural legacy. Recovering its model of decentralized stewardship could offer a strategic framework for modern urban regeneration, transforming stations into multifunctional hubs while preserving regional floristic identity, in alignment with contemporary European sustainability models.

## 1. Introduction

Urban green areas, commonly known as urban greenery, represent a central and often historical part of urban infrastructure. They are fundamental for supporting social interaction, psychological well-being, urban biodiversity, environmental sustainability, climate mitigation, stormwater management, improving air quality and adding to the overall esthetic and economic worth of city areas [1,2,3,4]. This role has been recognized by the United Nations since the 1990s, which identified urban greenery as one of the main instruments for enhancing sustainable development and environmental conditions in cities [5,6]. Similarly, European cities, including Italian municipalities and local communities, have promoted the importance of urban greenery since 1994 through the Aalborg Charter. Furthermore, since 2000, European countries have recognized, through the European Landscape Convention, urban green areas as a vital asset for social well-being and overall quality of life. Finally, new guidelines were recently introduced to restore natural habitats which also indirectly influenced the urban areas [7,8,9]. Accordingly, Italy has established and updated regulations for the development of urban greenery, provided guidelines for its management, and outlined initial strategies for sustainable planning [10].

A key component of these planning strategies is the selection of appropriate plant species, which is essential for the success of urban greening projects and the development of the ecosystem services outlined above, especially under the intensification of climate change effects [11]. Native plants represent a valuable and primary resource in landscaping due to their ability to provide greater intensity and diversity of ecosystem services such as air pollutant reduction, carbon services, water regulation, and health benefits compared with non-native plants [12]. They are also more likely to provide resources for urban fauna and increase biodiversity in urban green spaces [13]. Non-native plants, however, remain widely present and commonly used in urban landscapes, typically introduced intentionally through the global horticultural trade for their desirable esthetic qualities, physiological traits, and ability to adapt to diverse environmental conditions [14]. At the same time, several non-native plants can pose a severe threat when introduced into new geographical regions such as the Mediterranean Basin: their rapid expansion may deplete native biological diversity, alter soil nutrient dynamics and pollination networks, contribute to species extinction, and cause agronomic production losses, ultimately affecting entire ecosystems [15,16]. Urban areas represent a hotspot for non-native invasive plants, and Italian cities are extensively colonized and negatively affected by their presence; among which the most widespread are *Ailanthus altissima*, *Parthenocissus* spp., *Phytolacca americana*, *Robinia pseudoacacia*, and *Sorghum halepense* [17].

Careful and context-specific plant selection therefore remains a central challenge for any new green infrastructure project. This challenge extends to the expansion of low-impact mobility systems, which is equally crucial within the broader framework of sustainable urban development, especially given that urbanization is expected to increase in the near future. To transform this trend into an opportunity for growth, the development of urban greenery must be symbiotic with the expansion of public transport systems. In large and congested urban areas, railways play an essential role in ensuring liveability and productivity. Indeed, rail transport is environmentally friendly, with lower impacts than road and air transport for both passenger and freight services [18].

Railway areas, primarily found within urban areas in Europe, represent highly urbanized territories characterized by well-established transport and engineering infrastructure. However, these areas often include overgrown, abandoned, or neglected tracks and post-industrial spaces, which lead to the deterioration of the urban landscape [19]. Furthermore, railway corridors are also recognized as effective pathways for the dispersal of plant species, including invasive ones, due to the continuous soil disturbance and the movement of goods and passengers [20]. However, their spatial configuration, which is favourable for connecting different neighbourhoods, and their large available areas facilitated their reassessment and drove intensive redevelopment efforts.

One of the most notable examples is the High Line in New York, an elevated railway in Manhattan that operated from 1934 to 1980 and was originally used for the transport of goods. Since 2006, it has been transformed into an elevated park that weaves through the cityscape. Spontaneous vegetation that developed during the period of abandonment has been partially preserved, while perennial species have been introduced, ensuring year-round esthetic value and creating an ecological niche rich in biodiversity [21]. In Europe, a comparable initiative was implemented earlier in Paris, where the obsolete railway linking Paris and Vincennes was transformed between 1988 and 1993 into a green corridor known as the *Coulée verte René-Dumont*, commonly referred to as the *Promenade Plantée*. Following the original railway path, the park includes elevated sections, tunnels, and stretches at street level. It features both ornamental and spontaneous vegetation, with openings that provide panoramic views of the city [22,23]. In Italy, the *Nodo Verde* project in Bari (Apulia) is currently one of the major ongoing railway-area redevelopment initiatives, with urban greenery serving as a primary design criterion. The project aims to create a new integrated urban green landscape within the existing context and to provide the city with a new, well-equipped public space in an area historically divided by railway infrastructure, reconnecting different parts of the city [24,25].

In this context, the innovative concept of incorporating vegetation into railway area management was already present in Italy by the late 1800s [26,27], and in the early twentieth century it was applied through a large-scale and well-organized initiative, namely the *Concorso Stazioni Fiorite* (Flowery Stations Contest, FSC), which was implemented across numerous stations along the Italian railway network [28]. Despite the relevance of this first initiative involving greenery in Italian railway areas and its influence on the railway landscape throughout the twentieth century, information on the FSC has remained fragmented [29,30] and somewhat forgotten, lacking an organic and comprehensive review. Therefore, this study aims to provide the first comprehensive synthesis of the FSC experience, focusing on the 1911–1915 period. This time frame was specifically selected as it represents the complete original cycle of the contest, forming a distinct historical and documentary unit for reconstruction. Specifically, we (a) clarify the historical evolution of the FSC, including the cultural and organizational principles that shaped its role in the development of Italian railway station gardens and (b) examine the architectural styles and plant species proposed and planted through the initiative. Finally, we (c) assess the long-term legacy of the FSC by evaluating the persistence of station gardens more than a century after their establishment, and through the case study of Ripafratta (San Giuliano Terme, Pisa, Italy). The results obtained could provide new tools to promote a renewed, multifunctional role for plants in Italian railway stations.

## 2. Results and Discussion

### 2.1. The Historical Context and the FSC Development Across 1911–1915

The *Belle Époque*, spanning from the late 1800s into the early twentieth century, was a period of significant cultural, artistic, and technological advancement in Europe [31].

During this period, international exhibitions, also known as universal exhibitions, showcased a wide range of products, from advanced machinery and innovative technologies to traditional consumer goods such as textiles and furniture, produced using established methods. They also featured primary products such as minerals and agricultural crops, along with displays of a ‘social’ or ‘educational’ nature. The primary objective was to deliver an exhaustive depiction of the products and activities of the participating nations [32,33].

In 1911, Italy celebrated the fiftieth anniversary of its unification by hosting the International Exposition in Rome (EXPO 1911), which was part of a broader commemoration marking Italy’s three capitals: Rome (1870-present), Florence (1865–1870), and Turin (1861–1865) [34].

**Figure 1:**
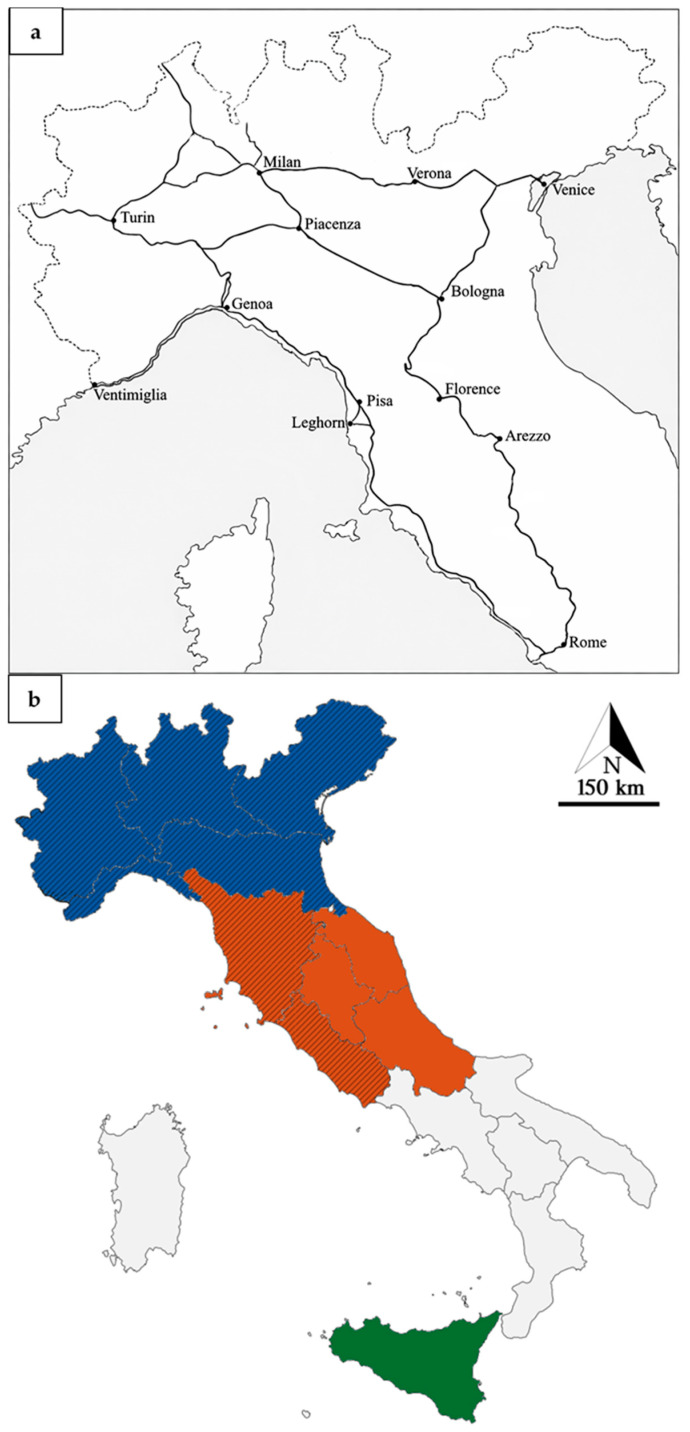
(**a**) Simplified map of Northern and Central Italy, based on an historic map showing the railway lines involved in the first FSC in 1911, available on the Emilia-Romagna Region website [37]. (**b**) Subdivision of Italy into 16 compartments, the forerunners of today’s regions, before the First World War. The compartments involved in the 1911 contest are marked with a line pattern; those involved in the 1912–1914 contests are shown in blue, and those in the 1913–1914 contests in red. The compartment used in a preliminary test in 1913 is shown in green. Grey compartments were never involved in the Flowery Stations Contest (FSC). Northern Italy was the main area of participation, followed by Central Italy. At the same time, contests in Southern Italy and the Islands were never held due to the outbreak of the First World War.

Following these events, *Touring Club Italiano* (Italian Touring Club, ITC), in collaboration with the *Federazione Italiana dei Consorzi Agrari* (Italian Federation of Agricultural Consortiums, IFAC), decided to promote an initiative aimed at improving the quality of railway stations, inspired by a similar event previously organized in France by the *Touring Club de France* [35]. The primary objective was therefore tourism-oriented, seeking to promote and showcase Italy to foreign visitors arriving to participate in the celebrations for the fiftieth anniversary of the Unification of Italy [36]. Thus, only the stations on the principal railways were involved, such as Florence, Milan, and Rome (Figure 1a).

The initiative was highly successful, prompting the organizers not only to repeat it the following year (1912), which was also supported by the FS (*Ferrovie dello Stato Italiane*, Italian State Railways), but also to expand it into a national project [38,39]. Thus, the competition was restructured, dividing Italy into three macro regions (North, Central, South and Islands), with each participating in a specific year. In detail, the North in 1912, Central in 1913, and South Italy and the Islands in 1914 [40]. Furthermore, new funds were allocated, increasing contributions to the management of the competition [39] (Table 1).

In 1912 and 1913, the editions were held in Northern Italy (Piedmont, Lombardy, Liguria, Veneto, Emilia) and Central Italy (Tuscany, Marche, Umbria, Lazio, Abruzzi and Molise; Figure 1b) [44]. To support the maintenance of the green areas of the stations that participated in the previous year, the second edition for Northern Italy in 1913 was held simultaneously with the first edition for Central Italy, and in 1914 the second edition for Central Italy was held concurrently with the third edition for Northern Italy [44,45,46].

In 1914, the first edition of the competition dedicated to Southern and Insular Italy was supposed to take place [43]. A preliminary approach had already been made in 1913 with a preparatory competition in Sicily, a sort of trial run for the larger event [42]. However, plans changed due to the outbreak of the First World War (WWI) in Europe in July 1914. The subsequent economic crisis linked to the conflict and the decline in tourism, despite Italy not yet being involved in the war, led to a change of plans. Moreover, both the FS and the IFAC disengaged from the competition. The new edition was cancelled and postponed to a later year, with a focus on reconfirmation competitions for Northern and Central Italy [47]. In 1915, no competition was held. The FS accepted the ITC’s suspension proposal in October 1914 [48].

### 2.2. FSC Aims: From Hygienic Improvements to Chemical Fertilization

The exaltation of Italy and its landscape for tourism purposes was the central objective of the FSC’s inaugural edition in 1911 [40]. The goal was to make train stations along the railway lines serving the 1911 Expositions more pleasant for both passengers stopping there and those merely passing through, who could observe the scenery from train windows [36]. In subsequent editions, the emphasis shifted to additional goals that had been secondary in the first iteration but later gained greater prominence. These included improving the hygienic and esthetic conditions of railway stations, promoting a culture of ornamental greenery and also of mineral-based chemical fertilization [45,49].

The stations’ hygienic and esthetic conditions were intrinsically tied to one significant issue, which was soot. At the time, trains were all steam-powered, and the particulate matter released by moving locomotives would cover platforms and station structures. While some stationmasters took proactive steps to clean these areas, many stations were plagued by layers of soot, creating significant discomfort and degradation. This issue was further exacerbated by other materials generated during loading and unloading, such as dust, discarded paper, wood shavings, and packaging [45]. Excessive crowding at stations was also criticized, though this concern reflected the elitist views of the period’s upper classes [45]. Overall, stations appeared barren, with little or no vegetation, except for occasional plantings introduced by individual stationmasters on their own initiative. The spaces served primarily functional purposes, with minimal attention given to liveability or esthetic quality [45].

Thus, to promote passion for ornamental plants and greenery among stationmasters, the population, and competition participants, various strategies were implemented, targeting both competition participants and individuals not directly involved in the initiative [36,49]. Participants were strongly encouraged to use perennial species to ensure that the ornamental improvements would be maintained over time, creating a lasting element of beauty that required ongoing care [49]. Additionally, the competition leveraged the pride of individual stationmasters by establishing a mixed system of rewards that included both monetary prizes and honorary recognition. To sustain interest and commitment, stationmasters were also encouraged to participate in subsequent editions [43]. Moreover, efforts were acknowledged in monthly publications, booklets, invitations to ITC members to send their photographs, and through a parallel photographic contest (at least in 1912) aimed at promoting the initiative to the general public [38,40,46,50,51,52]. At the end of each competition, summary reports highlighted the winners and described their gardens and the plants used, aiming to inspire imitation across the population, who could directly observe the esthetic improvements achieved through the rational cultivation of ornamental species [36,45,46].

From a commercial standpoint, the IFAC aimed to promote the rational use of mineral-based chemical fertilization, which was still scarcely adopted in Italy in the early twentieth century compared to other countries [53]. For this reason, the IFAC decided to provide participating stationmasters with free bags of fertilizer for use in the FSC, and it also considered their use as one of the evaluation criteria in the overall assessment carried out by the jury [41]. In 1912, the products supplied consisted of 10 kg of a mixture of mineral superphosphate and potassium sulfate, and 4 kg of sodium nitrate [49]. In addition, to support the rational use of mineral-based chemical fertilization by stationmasters, a brief explanatory text was included in the competition programme [49].

### 2.3. FSC Organization and Competition Ruleset

In the early twentieth century, Italy’s political landscape was predominantly shaped by liberalism, particularly during the Giolittian era (1900–1914), when political power remained largely in the hands of a restricted liberal elite drawn from the upper and middle-upper classes. At the same time, however, the period marked a gradual move toward greater democratization, most notably through the expansion of male suffrage in 1912 [54,55,56]. Likewise, the FSC reflected this transitional moment, marked by elite control on one side and expanding democratic inclusion on the other.

Stationmasters’ participation in the competition was open and voluntary, and they were formally invited to take part by ITC. Those who chose to participate were required to submit a form attached to the competition programme to the ITC headquarters [41,43,57]. Moreover, the stationmasters’ subordinates, who at the time constituted an essential workforce for the functioning of the railway system, were also involved in the initiative [44]. By engaging such a wide range of railway employees, the competition became a means of linking greenery to professional pride and to a shared sense of responsibility among staff, in a way that echoed the broader climate of gradual civic participation of the period.

In contrast, the organizing committee was composed of members of the middle and upper classes, reflecting the transitional phase of the Italian liberal political model in the early twentieth century, when universal suffrage had not yet become the norm.

The organizing committee had around 37 members, including knights, engineers, professors, commanders, officers, doctors, lawyers, and princes (often holding multiple titles) [41]. Examples include Vittorio Alpe and Guglielmo Körner, professors at the Higher School of Agriculture in Milan (now part of the University of Milan); Giberto Borromeo, industrialist and politician, from the well-known Milanese family; Ugo Brizi, botanist, mycologist, and director of the Brera Botanical Garden (Milan); Ludwig Winter, botanist and landscape architect; and Giuseppe Roda, a landscape architect working in Italy and abroad, who also worked for the Savoy royal family [41,58].

Above them, an executive committee composed of 12 members (selected from the broader group) oversaw the competition. The formal approval of the final ranking depended on this committee [41,59]. Moreover, the executive committee delegated the evaluation of the stations to a general jury appointed during the competition which was even composed of members of the organizing committee [41]. At the top of the organizational structure stood an honorary presidency, composed of three individuals with purely representative functions. In 1912, these were the Ministry of Agriculture, Commerce and Industry; the Ministry of Public Works; and the General Director of the FS [41].

The final ranking of the contest was determined indirectly by the general jury, which appointed specific jury delegates, both approved by the executive committee, who were required to follow precise instructions when preparing their reports, as outlined in the competition programme booklet [41]. The exact number of delegates is known only for 1913: 30 along the railway lines of Central Italy and 45 along those of Northern Italy [43]. They recognized the participant stations through a notice exposed by the stationmasters [40]. At the conclusion of the initiative each year, Giuseppe Roda was entrusted with writing a final report summarizing the results of the competition [36,41,60]. The competition concluded with an awards ceremony that highlighted the diversity of contributions and further reinforced the relationship between the participants and the event.

### 2.4. FSC Prizes Beyond Mere Competition

**Figure 2:**
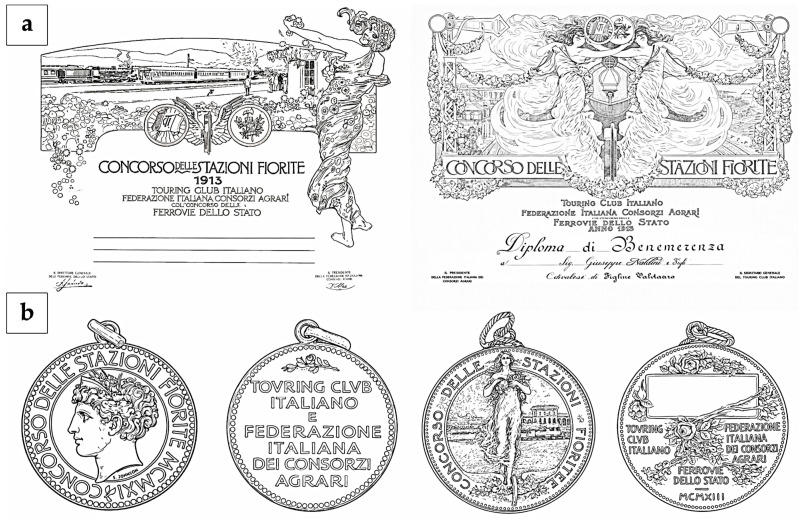
(**a**) Line art reconstruction based on two different types of the certificate awarded to stationmasters in the 1913 Flowery Stations Contest (FSC). The original template of the first certificate (left), authored by the artist Leopoldo Metlicovitz (1868–1944), is preserved by *Fondazione Massimo and Sonia Cirulli* archive. One certificate used for the Stresa station (Piedmont), bearing the signatures of Federico Johnson and Vittorio Alpe, heads of Italian Touring Club (ITC) and Italian Federation of Agricultural Consortiums (IFAC), respectively, was documented from a private collector market before 2023. Another version of the certificate, preserved privately by Mr. Guido Naldini used in 1913 for the Figline Valdarno railway station (Tuscany). All certificates featured colour typical of early twentieth-century illustrations, which has been omitted in the present line-art reconstructions. (**b**) Line art reconstruction based on medals given to stationmasters during the FSC. The first type (left) belonged to the 1911 contest, and it lacks the FS name on the reverse and features a simpler design than the 1913 medal (right). One copy of the 1911 medal and two copies of the 1913 medal were documented by private collector market before 2023. No other certificate versions or medal types were found in our research.

The awarding was based on three fundamental elements: a certificate of participation, a medal, and monetary compensation, and their combinations determined different merit classes for participating stations in relation to their greening performance. After the first contest edition in each macroarea, confirmation certificates were assigned to the participants that maintained vegetation presence and quality [41,46,52] (Figure 2).

**Figure 3:**
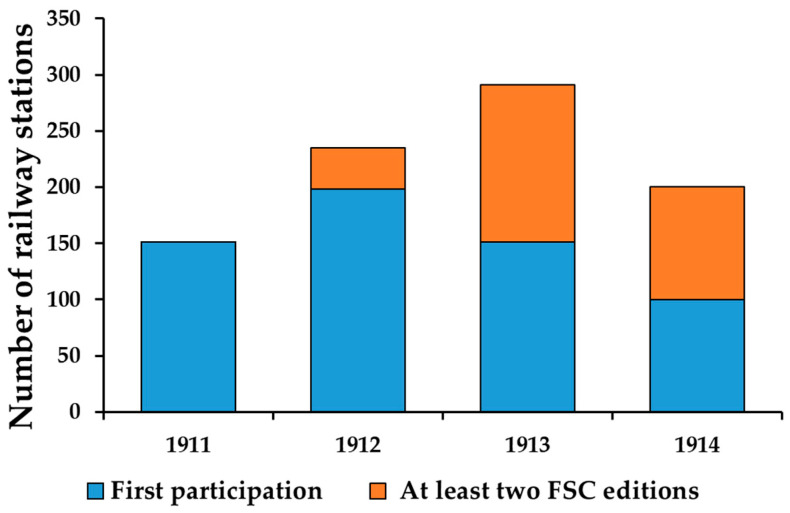
Number of railway stations participating in the Flowery Stations Contest (FSC) for the first time (blue) and in at least two FSC editions (orange), by year. The number of stations participating for the second time in a FSC in 1912 is limited to the station reported in the list of the awarded stations due to the lack of a complete data [38].

Medals were categorized as gold, gilt silver, silver, and bronze, with their distribution varying across the four FSC editions. Notably, gold medals were named after institutions, such as the Ministry of Agriculture, the Ministry of Public Works, the Lombard Horticultural Society, the Italian Farmers’ Society, and the Italian Hoteliers’ Society, while the gilt silver medal was offered and named after the Ministry of Agriculture, highlighting the institutional recognition of vegetated environments in railway spaces [41,52]. Monetary prizes were differentiated according to staff categories and performance levels, depending on the classification and the year (Stationmasters: 100/50 *Lire*, the Italian currency at the time; approximately 470 and 235 Euros in 2026; subordinates: 25/20/15 *Lire*, approximately 118, 94, and 71 Euros), further reinforcing incentives for maintaining and improving greenery within stations [41,52]. Over time, the system evolved with additional awards, including certificates of merit, commemorative medals for out-of-competition participants, and prizes sponsored by private companies. This flexible structure enabled broad participation across Italian railway divisions, with more than 150 stations involved annually and a peak of 291 in 1913 [43,44,52] (Figure 3).

The FSC framework favoured widespread engagement and the progressive integration of vegetation management into station practices rather than a strictly competitive logic, but the outbreak of WWI led to a decline in interest in the initiative, as national priorities shifted away from urban and environmental enhancement activities.

### 2.5. FSC Gardens’ Architectural Styles and Plants

Italian stations built until the early twentieth century were mostly characterized, aside from terminal stations and the major ones, by buildings with empty spaces surrounding them used as needed for railway activities, with a layout generally parallel to the railway lines.

All these elements together shaped the form of the gardens created by stationmasters during the FSC [29]. The arrangement of plants oriented clearly toward passengers on trains and those using the station, ideally recalling the idea of a *hortus conclusus*, whose boundaries are not physical walls but imaginary ones defined by the limits of the spaces dedicated to the functioning of the railway system, such as those observed in the 1911 and 1913 FSC photographs of Bordighera (Liguria) and Poggibonsi (Tuscany) [61,62]. Garden dimensions varied widely, from small plots to more extensive areas, depending on the station’s placement within the surrounding topography. In cases where the available station grounds were insufficient to accommodate a proper garden, floral arrangements were instead placed in hanging structures attached to the platform canopy and/or in raised flowerbeds constructed on the boarding platform as in 1912 and 1913 in Pontedecimo (Liguria) or in Dorio (Lombardy) [63,64]. Gardens followed either the Italian formal style, with regular boundaries and topiary, or the more naturalistic English style, as seen in Vasto (Abruzzi), Santa Margherita Ligure (Liguria), and Schio (Veneto), respectively, which also sometimes featured decorative elements such as fountains, statues, and vases [57,58,59]. Floral arrangements involving herbaceous plants, shrubs, woody climbers, and trees, were preferred by FSC organizers, as well as the use of perennial species to ensure the continued improvement of railway stations even after the end of the competition or the replacement of stationmasters [49].

**Figure 4:**
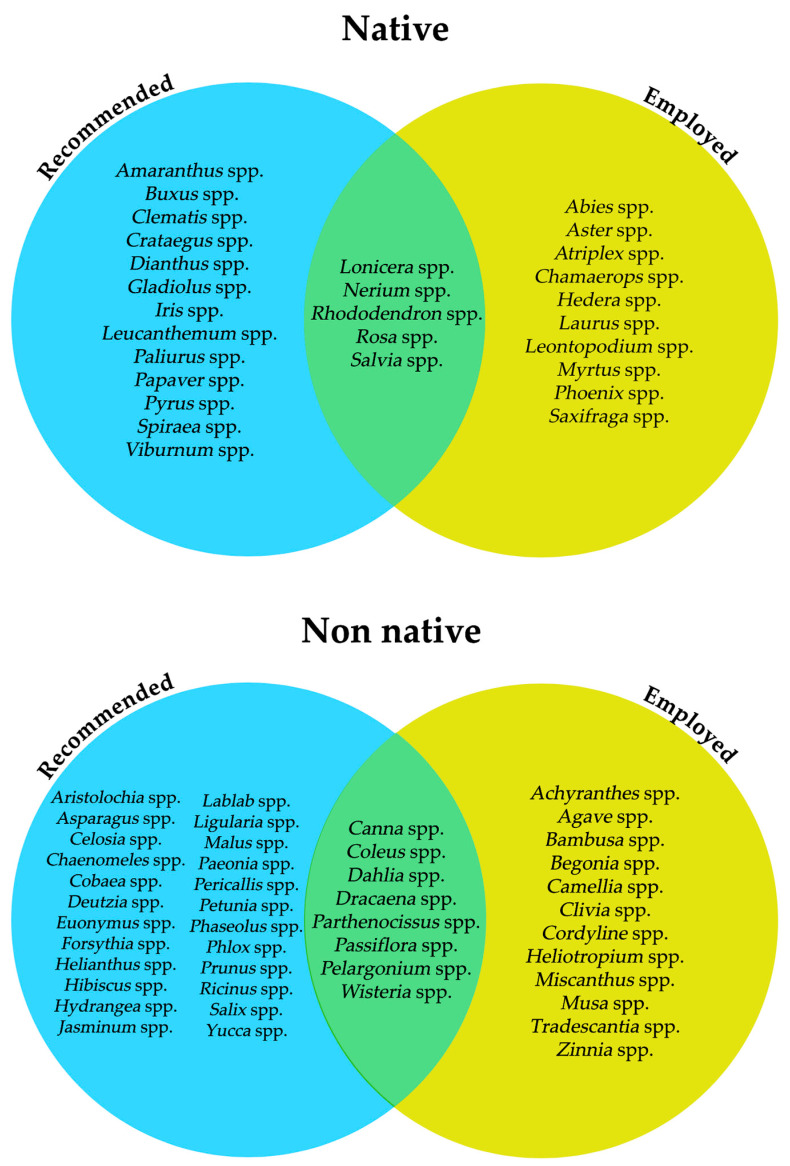
Venn diagrams reporting the plant genera recommended by the organizers of the Flowery Stations Contest (FSC), as listed in the booklet *Concorso delle Stazioni Fiorite. Le piante, i fiori, e i fertilizzanti. Consigli e suggerimenti per i concorrenti* (Flowery Stations Contest: The Plants, the Flowers, and the Fertilizers. Advice and guidelines for competitors), and the genera effectively employed by stationmasters during the contest, divided into native and non-native classifications [36,46,49,70]. Common genera are shown.

During the *Belle Époque*, exotic and profusely flowering species were highly appreciated in Europe, arriving from all over the world through the colonial trade networks, a process largely driven by Britain’s botanical institutions [65]. This trend can also be observed in the 1912 FSC booklet titled *Concorso delle Stazioni fiorite. Le piante, i fiori, e i fertilizzanti. Consigli e suggerimenti per i concorrenti* (Flowery Stations Contest. The plants, the flowers, and the fertilizers. Advice and guidelines for competitors), which was created to assist and guide the stationmasters in plant selection and arrangement, although the organizers also suggested taking inspiration from local gardens to identify plants better adapted to local conditions [49] (Table 2). Accordingly, stationmasters implemented non-native plants in the gardens of railway stations. Photographs of Schio station (Veneto) showed *Miscanthus sinensis* and *Musa bosjoo*, Nervi (Liguria) featured *Cordyline australis*, and *Phoenix canariensis*, Bordighera (Liguria) displayed *Agave* spp., *Clivia* spp., and *Pheonix canariensis*, and Santa Margherita Ligure (Liguria) included *Agave* spp., *Cycas* spp., *Cordyline australis,* and *Phoenix* spp. [61,66,67,68]. The analytical assessment carried out using the list of recommended genera confirmed the organizers’ preference for non-native plants, which accounted for more than half of the recommended genera (64%), whereas only 36% were native [49] (Figure 4). An interesting historical aspect of this list is the presence of *Phaseolus* spp. (specifically *P. coccineus*), traditionally cultivated as a food source but also for ornamental purposes, due to its abundant red flowers. Likewise, the assessment of the genera planted showed that more than half (57%) of those effectively implemented by stationmasters were non-native and more than half of them (60%) were selected without following the organizers’ recommendations. In contrast, the analysis also revealed that stationmasters employed a higher proportion of native plants (43%) than originally suggested, more than half of which (67%) had not been indicated in the booklet and belonged to genera such as *Abies*, *Aster*, *Laurus*, *Myrtus* and *Saxifraga* [36,46]. These data showed that, despite a general tendency toward floristic homogenization in plant use, due to the influence of the horticultural market of the period, the local selection implemented by stationmasters during the contest was oriented toward rebalancing the distribution between non-native and native genera. Although limited in scale, these choices demonstrated the capacity of each territory to offer native ornamental plants and the participants’ interest in employing and preserving them. This unconsciously anticipates a contemporary sensitivity toward biodiversity, showing how locally informed plant selections can counteract floristic uniformity and support regional floristic identity, especially in managed urban areas where plant diversity has historically declined [69]. Salbertrand station (Piedmont) represented a complete exception among the stations, aligning closely with what would now be considered a biodiversity preservation approach by employing exclusively native plants in order to adapt the garden to the low temperatures of the Alps. The jury endorsed the project’s intelligent and well-considered plant selection [36], though for reasons rooted in ornamental and acclimatization ideals rather than the sustainability and ecological concerns that drive contemporary landscape design. This is evidenced by the organizers’ inclusion of *Parthenocissus* spp. in their planting suggestions, a genus that today includes some of the most widespread non-native invasive ornamental plants in Italian cities [17] (Table 2). Since then, the approach to plant selection has shifted considerably. Native species are now increasingly preferred not only because of their well-established ecological benefits, including enhanced biodiversity, greater ecological stability, and reduced irrigation requirements, but also because research has demonstrated their ability to achieve ornamental performance comparable to that of non-native species [12].

### 2.6. The End of the FSC Experience and the Post-War Contest

**Figure 5:**
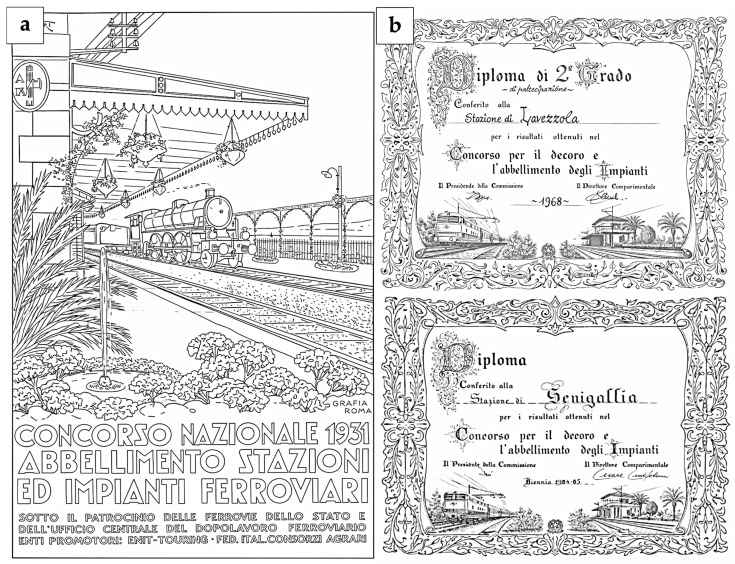
(**a**) Line art reconstruction based on a poster promoting the 1931 contest, preserved in *Museo Nazionale Collezione Salce* (National Museum Salce Collection, Treviso, Veneto), depicting hanging potted plants and ornamental shrubs on the platform near the station building; (**b**) two certificates awarded to the railway stations of Lavezzola (Emilia-Romagna) and Senigallia (Marche), from the minor contests held in 1968 and 1984–85, respectively, both illustrated with stylized shrubs and trees flanking the station buildings and railways. Original colours have been omitted.

The FSC experience ended with the escalation of WWI across Europe, including Italy. In 1925, the contest was brought up again for the Jubilee Year, with a slightly modified name, “*Concorso abbellimento stazioni*” (Station Beautification Contest), and under the organization of FS, ITC, and IFAC, together with the *Ente Nazionale Italiano per il Turismo* (Italian National Tourist Board; ENIT). Moreover, it was held on the same railway lines selected in 1911 [71]. In 1926, the contest was carried out primarily in Southern Italy and the Islands, covering for the first time those parts of the country that had been excluded in 1914 due to the outbreak of WWI [72]. Successive editions were organized during the 1930s and, following the Second World War (WWII), from the 1950s to the 1960s [73]. The latest ones were minor contests held during the 1970s and 1980s, as shown in several certificates from that period [74] (Figure 5), demonstrating that FSC’s influence persisted even after its conclusion and contributed to shaping Italian railway stations throughout the century.

### 2.7. Analytical Assessment of FSC Impact Across the Century

**Figure 6:**
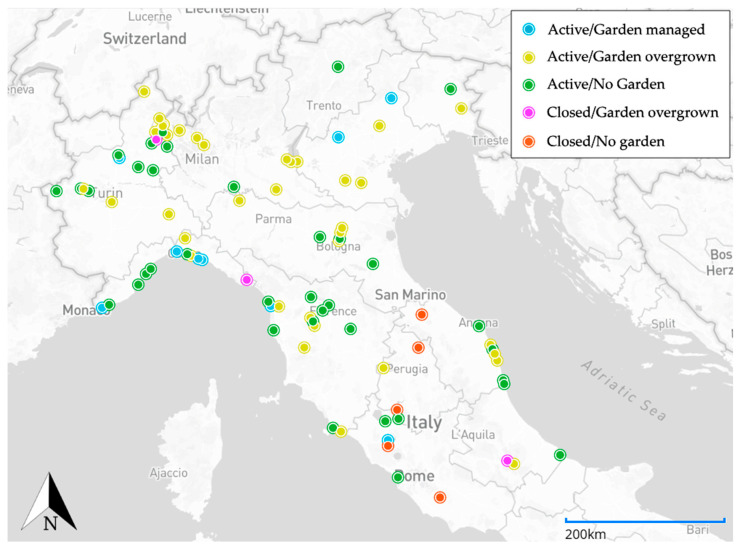
Railway stations included in the analytical assessment of the impact of the Flowery Station Contest (FSC) over the century. Stations were classified according to their operational status (active or closed), the presence of a garden, and garden condition (managed or overgrown). Other details are available in the Supplementary Table S2 and Supplementary Material S2.

**Figure 7:**
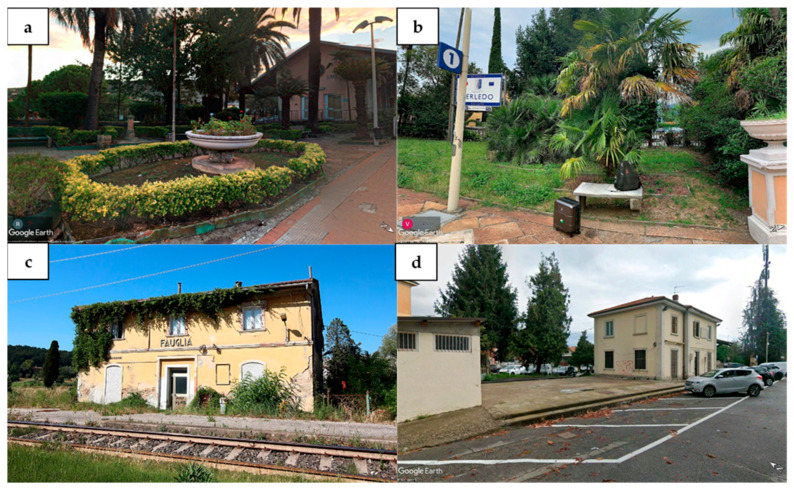
Several railway stations still retain garden areas that may have originated during the FSC. (**a**) Santa Margherita Ligure (Liguria) and (**b**) Varenna (Lombardy) display such garden areas, whereas they have completely disappeared in other locations, such as (**c**) Fauglia (Tuscany) and (**d**) Lurate Caccivio (Lombardy). This loss can be attributed to the operational demands of railway activities, limited maintenance, the progressive decline of the stationmaster’s role, and the closure of many minor stations associated with technological advancements [76,77].

The majority of the railway stations, among the 90 participating in the FSC between 1911 and 1913 that ranked within the first thirty positions, are still active (91%) (Figure 6; Supplementary Table S2; Supplementary Material S2). The remaining ones, all belonging to the minor structure category, were closed due to abandonment, closure of the railway line, or damage caused by WWII. More than half of the railway stations (54%), including two closed stations, still currently feature a garden, although only a few stations (18%) maintain them, whereas most display neglected and/or overgrown vegetation (82%), such as Santa Margherita Ligure (Liguria) and Varenna (Lombardy), respectively (Figure 7). Moreover, all stations in which gardens are still present belong to either the minor (63%) or medium (37%) structure categories, although their extent and condition vary considerably. These data suggest that stations located in rural areas were less affected by changes across the century, thus preserving both the original building structure and the garden area. In several cases, station gardens have completely disappeared (46%), such as in Fauglia (Tuscany) and Lurate Caccivio (Lombardy), due either to the construction of new station buildings or to the closure-related causes mentioned above. Although railway infrastructures have undergone major changes since the early twentieth century, including reduced maintenance of non-essential infrastructures, reorganization of railway functions and roles, and technological advancements, gardens still persist in many Italian railway stations, representing a trace of the FSC and its capacity to shape the image of railway stations across the century [75]. In our case, the progressive decline of station gardens is strictly associated with the gradual decline of the stationmaster’s role and presence [75]. The case study of Ripafratta railway station (San Giuliano Terme, Pisa, Tuscany) illustrates in detail all the dynamics highlighted by the data.

### 2.8. The Case Study of the Ripafratta Railway Station (San Giuliano Terme, Pisa, Tuscany)

**Figure 8:**
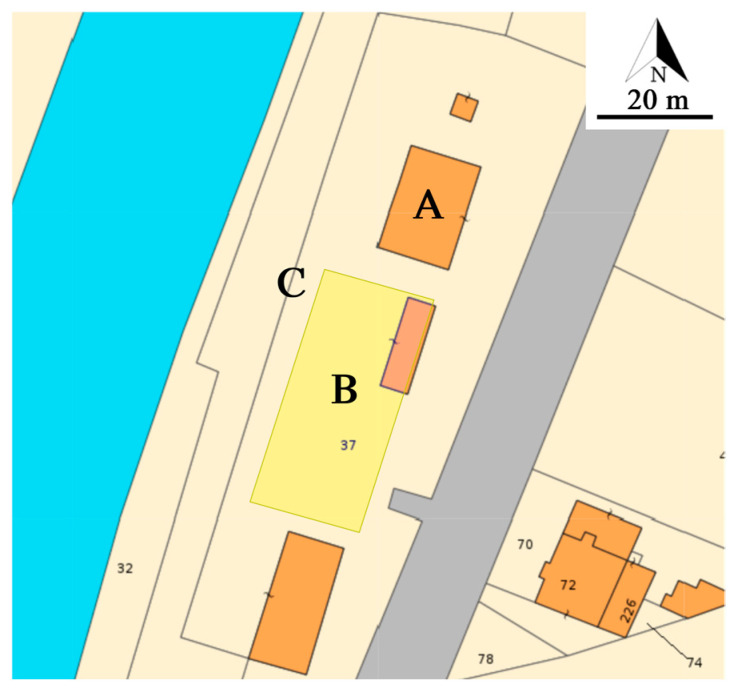
Ripafratta railway station (San Giuliano Terme, Pisa, Tuscany) as shown in the cadastral map (Cadastral Municipality: San Giuliano Terme-A562; Sheet 2, Parcel 37). (**A**) The station building, (**B**) the garden area (light yellow rectangle), and (**C**) the active Pisa–Lucca railway line [80].

Ripafratta railway station (43°49′18.12″ N, 10°24′57.6″ E; San Giuliano Terme, Pisa, Tuscany; 12 m a.s.l.) is a small station located on the railway line connecting the cities of Pisa and Lucca. It was inaugurated in 1846 and was part of one of the first international railway lines linking the Duchy of Lucca and the Grand Duchy of Tuscany [78]. The current building was constructed in 1909 and included (and still includes) several adjacent open areas used for train operations and management [79] (Figure 8).

**Figure 9:**
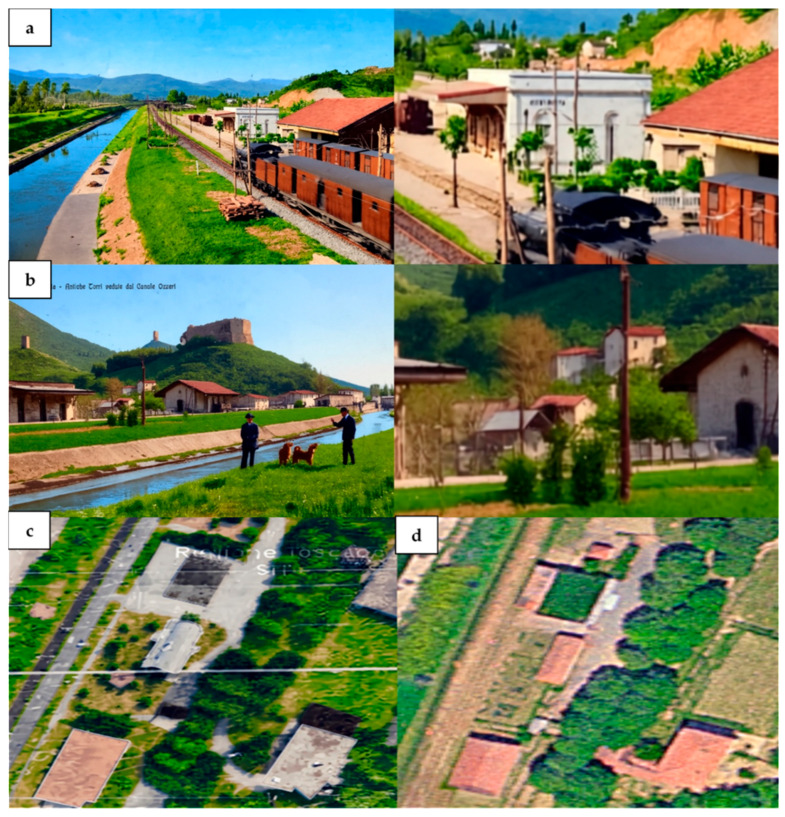
(**a**,**b**) Historical photographs of the Ripafratta railway station (San Giuliano Terme, Pisa, Italy Tuscany) from the first half of the twentieth century (left), with garden details (right), preserved by the local association *Salviamo La Rocca di Ripafratta*. (**c**,**d**) Aerial photographs from 1954 and 1965. All images were colourized using AI with the ImgUpscaler and Krea online tools to enhance garden visibility. Original photographs are available in Supplementary Figures S1 [30,84,85,86].

This station exemplifies the positive, lasting impact generated by the FSC. In 1913, following the first competition held in Central Italy, the stationmaster Olinto Gragnani took part in the contest with the support of local notables, such as Baroness De Virte, a local noblewoman passionate about ornamental plants [81,82]. Together, they likely converted the only free rectangular area (about 576 m^2^) parallel to the railway lines and located to the southwest of the main station building into a garden. Although scarce details are available regarding the garden’s layout or plant species (a general wide use of flowering coloured ones) during this period, the station garden won a silver medal, a certificate, and 100 *Lire* in the contest (approximately 470 Euros in 2026) [44,52,82]. FSC participation influenced the configuration of the station area in the subsequent decades. Photographs taken between the 1920s and 1930s show that the garden was still present, confirming its location and revealing the presence of shrubs, some young or small woody species, and possibly a surrounding fence. After WWII, in 1954, an aerial photograph of the same area showed several small plants, regularly arranged in relation to the station buildings, as well as a circular feature likely identifiable as a fountain. Between 1954 and 1965 (around 1960, based on an image from the FS-archive), the garden was completely redesigned. An aerial photograph from 1965 clearly shows the creation of four variably sized square flowerbeds aligned along the southwest side, bordered by hedges and interspersed with walkways [30,83,84] (Figure 9).

**Figure 10:**
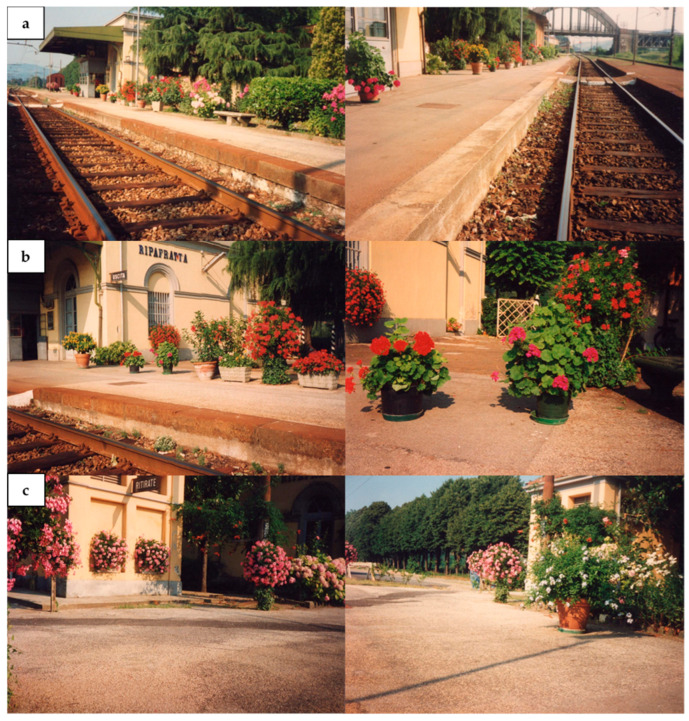
Photographs of the Ripafratta railway station (San Giuliano Terme, Pisa, Tuscany) from the late 1980s and early 1990s, preserved in the private collection of Mr. Lido Pardi of the local association *Salviamo La Rocca di Ripafratta*, were taken during the final period of management by stationmaster Antonietta De Benedictis. (**a**) *Cedrus deodara*, *Thuja plicata* and topiary *Prunus laurocerasus* were associated with *Rosa* spp., *Hydrangea* spp., and other flowering plants in pots placed alongside the train platform in the managed garden area. The photographs were taken from the southern (left) and northern (right) parts of the station. (**b**) Details of the potted flowers. *Camellia japonica*, *Pelargonium* spp., and *Pelargonium peltatum* were visible, the latter also used along the window edges. (**c**) Details of the northern part of the station, also decorated with flowering plants. *Campsis radicans*, *Hydrangea* spp., *Plumbago capensis*, and *Pelargonium peltatum* can be recognized on raised supports and on the windowsills of the station building.

This garden structure has been preserved and is visible over the decades in the online aerial photograph database of the Tuscany Region. Its layout is still visible on the ground today [84]. The hedges were probably composed of topiarized *Prunus laurocerasus*, while, from northwest to southeast, the flowerbeds alternated between *Cedrus deodara* and *Thuja plicata* in two repeated sequences. Their presence is confirmed by partially visible photographs of the garden dating from the 1960s to the early 1990s conserved in the *Fondazione FS*—Italian State Railways Foundation archive, in the book *Un treno per Lucca. Ferrovie e tranvie in Lucchesia, Valdinievole e Garfagnana. Funicolare di Montecatini* by Adriano Betti Carboncini, and in the private collection of Mr. Lido Pardi, a member of a local association named *Salviamo La Rocca di Ripafratta* [30,78,83] (Figure 10). Moreover, in a tree census carried out in 2019, two *C. deodara* trees were registered showing considerable dimensions (height ≥ 10 m; trunk diameter ≥ 0.4 m), consistent with the expected development of this species since the 1960s, considering its growth in a restricted space [30,87]. Similarly, two *T. plicata* trees were reported, showing reduced growth likely due to competition with *C. deodara* and its canopy shade, which are known to restrict *T. plicata* development [88]. Additionally, overgrown hedges of *P. laurocerasus,* forming large masses, were present [30].

**Figure 11:**
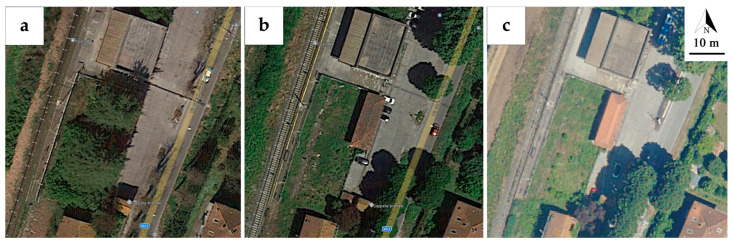
Satellite images and orthophoto of the Ripafratta railway station (Tuscany) and its garden show (**a**) the canopies of the two *Cedrus deodara* in the central part of the garden, as well as the overgrown *Prunus laurocerasus* hedges in the western and southern areas before the removal of all vegetation (3 April 2020); (**b**) the area after vegetation removal (8 October 2020), where the sidewalks and the garden’s original layout became visible once the plants were cleared; and (**c**) the area in 2025, which remains abandoned and empty [77,84].

Between 1971 and 1992, the gardens were well managed by the stationmaster Antonietta De Benedictis, one of the first women to hold this role in Italy. The garden reached its peak during these years and was awarded a prize for beautifying the station [89]. Images from 1992 confirm not only the above-mentioned species, but also the presence of *Hydrangea* spp. and *Rosa* spp. within the hedges alongside *Prunus laurocerasus*, as well as *Camellia japonica*, *Pelargonium* spp., *Pelargonium peltatum*, and other unidentified potted plants (Figure 9). Plants were located not only close to and within the garden area but also along the platform, extending to the northern side of the station. In this area, *Pelargonium peltatum* was positioned on raised supports and on the windowsills of the station building, as well as *Plumbago capensis* in pots, and *Campsis radicans* and *Hydrangea* spp., probably growing in flowerbeds. In 1993, the stationmaster position was abolished due to technological advances in railway management, and the station remained operational but unstaffed, as was the case for many other minor stations [75]. The place remained in use but was severely neglected, including the garden, and concerns about its improper use increased [90,91]. *C. deodara*, *T. plicata*, and overgrown *P. laurocerasus* were still present until 2020, alongside spontaneously established plant species, but were completely removed in the same year. Currently, the area is empty, and only the concrete flowerbeds and walkways remain [84] (Figure 11).

Despite the current situation, the history of Ripafratta railway station highlights the influence of the FSC across the century, with the garden consistently maintained through successive generations of stationmasters, demonstrating the positive long-term impact of greenery on public areas. This raises the question of whether some elements of the FSC experience could be reconsidered within a modern framework as a strategy to support economic, social, and environmental regeneration in station areas.

### 2.9. Future Perspectives for the Italian Railway Stations

In recent years, the EU has paid increasing attention to the unique role of railway stations within the urban landscape, rethinking stations as new multifunctional hubs for citizens, also through nature-based solutions [92]. Thus, greenery at railway stations and, more generally, in railway areas represents a valuable instrument that, when integrated with others, can generate urban renewal, sustainability, public health, and social inclusion. Currently, many important renewal projects, such as *Binari senza tempo*, have been and are being carried out on Italian railway lines, involving historic routes and buildings to preserve and enhance these assets through the sharing and participation of local populations and associations [93,94]. Additionally, in recent years, projects incorporating greenery have been developed at stations in major Italian cities, such as *Bari Centrale* (Bari, Apulia), *Roma Termini* (Rome, Lazio), and *Milano Tibaldi Università Bocconi* (Milan, Lombardy) [24,25,95,96].

However, a widespread national project that systematically addresses greenery as a tool for small, medium, and large stations is still lacking. A coordinated and diffuse system could ensure a more balanced territorial coverage, extending interventions beyond major urban centres into peripheral and provincial areas. The recovery of elements of the FSC, particularly its model of locally distributed governance involving station staff and communities, and its structured system of periodic evaluation and incentives, may offer an opportunity to achieve this objective, while simultaneously preserving historical memory, connecting past and present, and supporting the evolving multifunctional role of railway stations. Integrating this component could be essential for strengthening the capacity to regenerate marginalized areas and for providing significant environmental and ecological benefits across Italy, including mitigation of the urban heat island effect, reduction of air and noise pollution from train operations, carbon sequestration, creation of habitats for urban wildlife, and biodiversity conservation [97,98,99,100]. In addition, involving local communities, including vulnerable groups, in the management of station gardens may enhance collective ownership, reduce perceptions of insecurity, and support long-term maintenance. Such engagement can also strengthen social cohesion, reduce isolation, and improve overall quality of life [101,102]. These approaches have already been implemented in the United Kingdom by the organization Community Rail Network, with concrete results in reconnecting local communities and train operators in railway areas through greenery, while enhancing biodiversity, environmental sustainability, and community well-being [103].

## 3. Conclusions

In this study, a comprehensive reconstruction of the FSC initiative (1911–1915) and its legacy was carried out. Historical evidence shows (a) that the FSC involved broad railway station participation across northern and central Italy with the aim of promoting tourism and Italy’s landscape, addressing esthetic and hygienic issues such as locomotive soot, and fostering a broader cultural appreciation for ornamental greenery. The organization of the contest reflected the liberal ideas and the social structure of Italian society in the early twentieth century, reflecting a transition between elite control and expanding democratic inclusion, which is evidenced by a prize system that allowed for broad distribution among participants and highlighted that the objectives and participation in the contest went beyond mere competition. Although the FSC experience ended with WWI, it was revived in the following decades and continued to influence the design of Italian railway stations until the 1980s. The FSC station gardens (b) reflected the garden architectural styles and plant preferences of the early twentieth century. While exotic and flowering species were highly appreciated and recommended by organizers (accounting for 64% of suggested genera), the stationmasters’ initiative resulted in an increased usage of native plants, which reached 44% of effectively employed genera, including genera that were not recommended but were considered useful for the preservation of local biodiversity. This contrast between exotic and native plants highlights how the Italian railway landscape was shaped both by centralized esthetic visions and by deep connection of railway workers with their own territory. An analytical assessment (c) of 90 railway stations ranked in the top positions between 1911 and 1913 showed that, after more than a century, 54% still retained the established gardens, although only a few of them are currently managed, while a larger number are neglected. Additionally, all the stations belonged to the small- or medium-sized category, highlighting that stations located in rural areas were less affected by changes over time. However, the survey also revealed that 82% of surviving gardens are currently neglected or overgrown, a condition strictly associated with the progressive decline of the resident stationmaster’s role. The case of Ripafratta railway station is a clear example of the impact of the FSC over the twentieth century and of the changes that influenced the evolution of station gardens. Established in 1913, the garden was maintained for decades and reached its management peak during the tenure of the last stationmaster (around 1980s) after which a gradual decline began. Currently, the garden area is still physically present, although all vegetation was removed in 2020 and the site is now in a neglected condition.

The evidence confirms that the FSC persistently shaped the Italian railway landscape, leaving physical traces that endure despite the loss of local management and technological advancements. Its knowledge could provide new insights for the regeneration of railway station areas through plant-based solutions, helping to align Italian railway stations with emerging EU models of multifunctional hubs and generating positive outcomes for both local communities and rail operators. Preserving the social and environmental legacy of this initiative may also inform future policies for the renewal of Italian railway stations and support efforts to regenerate neglected urban areas.

## 4. Materials and Methods

A corpus of documents reporting official data about the FSC and the FSC at Ripafratta railway station was created between 2019 and 2026 through a systematic collection and organization of documentary sources. A table using Microsoft Excel 2016 (Microsoft, Redmond, USA) was constructed as a relational dataset in which each retrieved document represented a single record. For each record, bibliographic and archival metadata (archive or repository, document, source type, quantity of materials recovered from each document; Supplementary Table S1) were reported. Documents included: (1) award lists, (2) books, (3) booklets, (4) certificates, (5) journal articles and/or editorial notes, private documents, (6) a single master‘s thesis, (7) a single map, (8) medal images, (9) photographs, (10) reports, and (11) satellite images.

Published journal articles and editorial notes were retrieved from the digital archive of the *Rivista mensile del Touring Club Italiano* (Monthly Journal of the Italian Touring Club) for the years 1910–1915 (Italian Touring Club, ITC; https://www.digitouring.it), while bibliographical materials were consulted at the Central National Library of Florence, the National Library of Potenza and the libraries of the University of Pisa. Historical photographs of the contest were retrieved from the online archives of the Italian Touring Club (https://www.digitouring.it) and “*Fondazione FS”* (https://www.archiviofondazionefs.it; Italian State Railways Foundation). Furthermore, other materials (e.g., aerial photos, graphic images) were found on various websites (e.g., Ebay, https://www.ebay.it; Foundation Massimo e Sonia Cirulli, https://fondazionecirulli.org; Geoscopio, https://www.geosocopio.it). To achieve this result, multiple combinations of “*Stazioni fiorite*”, “*Concorso*”, “*Touring Club Italiano*”, “*Federazione Italiana dei Consorzi Agrari*”, “*Ferrovie dello Stato*”, “1911”, “1912”, “1913”, “1914”, “1915” were used as main keywords. In total, 81 FSC-only and Ripafratta railway station participation documents were collected (Supplementary Table S1).

Each retrieved document was independently examined to identify information relevant to the research objectives. Data were screened to find the following information: (i) contest organization; (ii) contest objectives; (iii) garden design; (iv) plant species recommended by organizers; (v) plant species employed by stationmasters; and (vi) contest outcomes. The same analytical framework was subsequently applied to the Ripafratta railway station case study to reconstruct its participation in the contest and its garden characteristics using all available documentary evidence.

Plant data organized in a table using Microsoft Excel 2016 (Microsoft, Redmond, USA) were used for complementary analysis. The plant taxa officially recommended by the FSC organizers were retrieved from the booklet *Concorso delle Stazioni fiorite. Le piante, i fiori, e i fertilizzanti. Consigli e suggerimenti per i concorrenti* [49] and then were compared with those effectively employed by stationmasters in the horticultural practice, as reported by the sources. Plants of the World Online tool [70] was used to standardize taxonomic names according to currently accepted botanical nomenclature, following the standards of the Royal Botanic Gardens, Kew. Taxa were reported at family, genus or species level depending on the level of identification provided in the original historical sources. With the same online tool, the biogeographical origin of each genus was assessed by distinguishing native and non-native ones on the basis of the Italian flora to assess the influence of the horticultural market of the period on local biodiversity. Native ranges were reported according to the World Geographical Scheme for Recording Plant Distributions (TDWG; https://www.tdwg.org/), using Level 1 (continental scale) geographic standardization [70].

A survey of railway stations was carried out to assess the long-term legacy of the contest. To evaluate the persistence of station gardens more than one century after the FSC, a representative sample of 90 railway stations participating in the editions held between 1911 and 1913 was selected. For each year, the first thirty ranked stations were selected. When a station appeared in more than one annual ranking, it was replaced by the next ranked station not previously selected in order to maximize the number of independent case studies. Railway stations were subsequently classified according to the historical FS/RFI (Italian State Railways/Italian Railway Network) classification system adopted until 2018 [Gold (Main structure), Silver (Medium structure), and Bronze (minor structure) categories], as it better reflects the historical and infrastructural relevance of stations, whereas the current RFI classification system is mainly based on operational and service-management criteria [104]. Geographical localization was ensured through Google Earth Pro [77] (Supplementary Material S2).

For each station, the following variables were recorded in a table using Microsoft Excel 2016 (Microsoft, Redmond, USA): year of participation in the FSC, contest ranking, current station name, operational status (active or closed), geographical region, presence or absence of a garden, apparent maintenance condition (managed or neglected), and any additional notes (e.g., abandonment, damage, reconstruction, station or line closure). Those data were obtained through visual assessment of satellite imagery and street-level photographs available through Google Earth Pro [77], complemented by information on the *Rete Ferroviaria Italiana*—RFI website [104], a company of the *Ferrovie dello Stato Italiane* (Italian State Railways, FS), and online resources [105,106] (Supplementary Table S2).

All tables related to plant taxa and railway stations were analyzed descriptively using Microsoft Excel 2016 (Microsoft, Redmond, USA), through comparison and calculation of percentages. This analysis was used to compare the plant taxa recommended by the FSC organizers with were those effectively employed by stationmasters, including their biogeographical origin (native vs. non-native), so as to evaluate the influence of the horticultural practices of the period on local plant biodiversity, and to assess the long-term physical persistence and conservation status of station gardens in relation to station classification.

## Figures and Tables

**Table 1:** Flowery Stations Contest (FSC) prize pool.

Year	Prize Pool Dedicated to Certificates and Medals (*Lire*/Euro ^a^)	Monetary Prize Pool (*Lire*/Euro ^a^)	Total Prize Pool (*Lire*/Euro ^a^)	Contributors	Reference
**1911**	2000/9483	4000/18,966	6000/28,449	IFAC, ITC	[39]
**1912**	3000/14,096	7000/32,891	10,000/46,987	IFAC, FS, ITC	[41]
**1913**	3500/16,412	8500 ^b^/39,859	12,000/56,272	IFAC, FS, ITC	[42]
**1914**	2500 */11,723	500/2344	3000 */14,067	ITC	[43]
**Total**	8500/39,991	20,000/91,716	31,000/145,775		

^a^ = value in € at 2026; ^b^ 500 *Lire* were given to the *Associazione del Bene Economico della Sicilia* (Association for Sicily’s Economic Welfare) for a short trial in preparation for the future competition in Southern Italy. * = Estimated from the total contribution of 31,000 *Lire* reported by [44] for the years 1911–1914. IFAC= Italian Federation of Agricultural Consortiums; FS = *Ferrovie dello Stato Italiane*, Italian State Railways; ITC = Italian Touring Club.

**Table 2:** List of plant phyla, families, genera, and species recommended by organizers and effectively employed by the stationmasters during the Flowery Stations Contest (FSC) organizers to stationmasters, as reported in the booklet *Concorso delle Stazioni fiorite. Le piante, i fiori, e I fertilizzanti. Consigli e suggerimenti per i concorrenti* (Flowery Stations Contest. The plants, the flowers, and the fertilizers. Advice and guidelines for competitors) [49] and by available sources [36,46,61,66,68], respectively. Asia = temperate and tropical parts; Asia-Te = temperate part; Asia-Tr = tropical part, GP = Grouping planting; FD = Flowering displays; HB = Hanging baskets; H = Hedge; SP = Solitary planting; VP = Vertical planting.

Plant Taxon	Italian Common Name	English Common Name	Arrangement	Life Form	Native Distribution (TDWG Level 1)	Reference
*Abies* spp.	*Abete* ^a^	Fir	SP	Tree	Africa, Asia, Europe, North America	[36]
*Achyranthes* spp.	*Achirante*	Chaff flower	FD	Herbaceous	Africa, Asia, Australasia, Pacific, South America	[36]
*Agave* spp.	*Agave*	Agave	SP	Herbaceous	North America, South America	[61]
*Amaranthus* spp. *	*Amaranto*	Pigweed	FD	Herbaceous	Asia, Africa, Europa, North America, South America	[49]
Arecaceae **	*Palme* ^a^	Palms	SP	Tree	Asia, Africa, Australasia, North America, South America	[36,49]
*Aristolochia macrophylla* *	*Aristolochia a foglie grandi*	Dutchman’s pipe	VP	Climber	North America	[49]
*Asparagus* spp.	*Asparagina*	Asparagus	HB	Herbaceous	Africa, Asia, Australasia, Europe	[36]
*Asparagus densiflorus* cv.*‘Sprengeri’* *	*Asparagina sprengeri*	Asparagus fern	HB	Herbaceous	Africa	[49]
*Aster* spp.	*Astri* ^a^	Aster	FD	Herbaceous	Asia, Europe, North America	[36]
*Atriplex* spp.	*Atroplice* ^a^	Saltbush	H	Shrub	Africa, Asia, Australasia, Europe, North America, Pacific, South America	[46]
Bambusoideae **	*Bambù* ^a^	Bamboo	GP	Herbaceous	Africa, Asia, Australasia, North America, South America	[36,49]
*Bambusa* spp.	*Bambuse* ^a^	Bamboo	GP	Herbaceous	Asia, Australasia	[36]
*Begonia* spp.	*Begonia* ^a^	Begonia	FD	Herbaceous	Africa, Asia, Northern America, South America	[36]
*Buxus sempervirens* *	*Bosso comune*	Boxwood	H	Shrub	Africa, Asia-Te, Europe	[49]
*Camellia japonica*	*Camelia* ^a^	Camellia	SP	Shrub	Asia-Te	[36]
Canna indica **	*Canne d’India* ^a^	Canna lily	FD	Herbaceous	North America, South America	[36,49]
*Canna* spp.	*Canna* ^a^	Canna lily	FD	Herbaceous	North America, South America	[36]
*Celosia* spp. *	*Creste di gallo* ^a^	Cockscomb	FD	Herbaceous	Africa, Asia-Tr, North America, South America	[49]
*Chaenomeles japonica* *	*Cotogno del Giappone* ^a^	Japanese quince	H	Shrub	Asia-Te	[49]
*Chamaerops humilis*	*Palma di San Pietro*	Mediterranean fan palm	SP	Shrub	Africa, Europe	[36]
*Clematis* spp. *	*Clematide*	Clematis	VP	Climber	Africa, Asia, Europe, North America, South America	[49]
*Clematis vitalba* *	*Vitalba* ^a^	Traveller’s joy	VP	Climber	Africa, Asia, Europe	[49]
*Clivia* spp.	*Clivia*	Clivia	FD	Herbaceous	Africa	[61]
*Cobaea scandens* *	*Cobea rampicante*	Cup-and-saucer vine	VP	Climber	North America	[49]
*Coleus* spp. **	*Coleo*	Coleus	FD	Herbaceous	Africa, Asia-Tr, Australasia	[36,49]
*Cordyline australis*	*Cordilinea*	Cabbage tree	SP	Shrub	Australasia	[67]
*Cordyline* spp.	*Cordilinea* ^a^	Cordyline	SP	Shrub	Asia, Australasia, Pacific, South America	[36]
*Crataegus monogyna* *	*Biancospino* ^a^	Common hawthorn	H	Tree/Shrub	Africa, Asia-Te, Europe	[49]
*Crataegus* spp. *	*Biancospino*	Hawthorn	H	Tree/Shrub	Asia-Te, Europe, North America	[49]
*Dahlia* spp. **	*Dalia* ^a^	Dahlia	FD	Herbaceous	North America, South America	[36,49]
*Deutzia* spp. *	*Deutzia* ^a^	Deutzia	SP	Shrub	Asia, North America	[49]
*Dianthus chinensis* *	*Garofano* ^a^	China pink	FD	Herbaceous	Asia	[49]
*Dracaena* spp. **	*Dracena* ^a^	Dracaena	SP	Tree/Shrub	Africa, Asia-Tr, Australasia, North America, South America	[36,49]
*Euonymus japonicas* *	*Fusaggine* ^a^	Japanese spindle	H	Shrub	Asia-Te	[49]
*Forsythia viridissima* *	*Forsizia*	Forsythia	SP	Shrub	Asia-Te	[49]
*Gladiolus* spp. *	*Gladiolo* ^a^	Gladiolus	FD	Herbaceous	Africa, Asia-Te, Europe	[49]
*Hedera* spp.	*Edera* ^a^	Ivy	VP	Climber	Asia-Te, Europe, Europe	[46]
*Helianthus* spp. *	*Girasole*	Sunflower	FD	Herbaceous	North America	[49]
*Heliotropium arborescens*	*Fior di vaniglia*	Heliotrope	FD	Herbaceous	South America	[36]
*Hibiscus syriacus* *	*Malvone delle Siria* ^a^	Rose of Sharon	H	Tree/Shrub	Asia-Te	[49]
*Hydrangea* spp. *	*Ortenisa a fiori in pannocchia* ^a^	Hydrangea	SP	Shrub	Asia-Tr, North America, South America	[49]
*Ipomoea indica* *	*Campanella perenne*	Blue morning glory	VP	Climber	North America, South America	[49]
*Ipomoea purpurea* *	*Convolvulo d’America* ^a^	Morning glory	FD	Herbaceous	North America, South America	[49]
*Iris* spp. *	*Giaggiolo* ^a^	Iris	FD	Herbaceous	Asia, Africa, Europe, North America	[49]
*Jasminum nudiflorum* *	*Gelsomino giallo* ^a^	Winter jasmine	VP	Climber	Asia-Te	[49]
*Lablab purpureus* *	*Dolico egiziano*	Hyacinth bean	FD	Climber	Africa, Asia-Tr	[49]
*Laurus nobilis*	*Lauro* ^a^	Bay laurel	H	Shrub	Africa, Europe, Asia-Te	[36]
*Leontopodium nivale subsp. Alpinum ^b^*	*Edelwiss* ^a^	Edelweiss	FD	Herbaceous	Europe	[36]
*Leucanthemum maximum* *	*Margherita gigante*	Shasta daisy	FD	Herbaceous	Europe	[49]
*Ligularia dentate* *	*Ligularia dentata*	Ligularia	FD	Herbaceous	Asia-Te	[49]
*Lonicera caprifolium* *	*Caprifoglio comune*	Italian honeysuckle	VP	Climber	Asia-Te, Europe	[49]
*Lonicera* spp.	*Caprifoglio* ^a^	Honeysuckle	VP	Climber	Africa, Asia, Europe, North America	[36]
*Malus domestica* *	*Melo* ^a^	Apple	SP	Tree	Asia-Te	[49]
*Miscanthus sinensis*	*Eulalia*	Chinese silver grass	FD	Herbaceous	Asia	[68]
*Musa basjoo*	*Banano giapponese*	Japanese banana	SP	Herbaceous	Asia-Te	[68]
*Myrtus communis*	*Mirto* ^a^	Myrtle	SP	Shrub	Africa, Asia-Te, Europe	[36]
Nerium oleander **	*Oleandro/Leandro* ^a^	Oleander	SP	Tree/Shrub	Africa, Asia, Europe	[36,49]
*Paeonia suffruticosa* *	*Peonia arbore* ^a^	Tree peony	SP	Shrub	Asia-Te	[49]
*Paliurus spina-christi* *	*Marruca* ^a^	Christ’s thorn	H	Shrub	Asia-Te, Europe	[49]
*Papaver somniferum* *	*Papavero* ^a^	Opium poppy	FD	Herbaceous	Asia-Te, Europe	[49]
*Parthenocissus quinquefolia*	*Vitivergine* ^a^	Virginia creeper	VP	Climber	North America	[46]
*Parthenocissus tricuspidate* *	*Vite del Canadà* ^a^	Boston ivy	VP	Climber	Asia-Te	[49]
*Parthenocissus* spp.	*Vitivergine* ^a^	Virginia creeper	VP	Climber	Asia, North America	[46]
*Passiflora caerulea* *	*Fiore della passione*	Blue passionflower	VP	Climber	South America	[49]
*Passiflora* spp.	*Passiflora*	Passionflower	VP	Climber	Asia, Australasia, North America, Pacific, South America	[46]
*Pelargonium grandiflorum* *	*Geranio imperiale*	Regal pelargonium	FD	Herbaceous	Africa	[49]
*Pelargonium peltatum* *	*Pelargonio a foglia d’edera* ^a^	Ivy-leaved pelargonium	FD	Herbaceous	Africa	[49]
*Pelargonium* spp. **	*Geranio/Pelargonio* ^a^	Geranium/Pelargonium	FD	Herbaceous	Africa, Asia-Te, Australasia	[36,49]
*Pericallis* spp. *	*Cineraria* ^a^	Cineraria	FD	Herbaceous	Africa	[49]
*Petunia* spp. *	*Petunia* ^a^	Petunia	FD	Herbaceous	South America	[49]
*Phaseolus coccineus* *	*Fagiolo di Spagna*	Runner bean	FD	Herbaceous	North America	[49]
*Phlox* spp. *	*Flogo*	Phlox	FD	Herbaceous	Asia-Te, North America	[49]
*Phoenix canariensis*	*Palma delle Canarie*	Canary Island date palm	SP	Tree	Africa	[66]
*Phoenix* spp.	*Phoenix* ^a^	Date palm	SP	Tree	Africa, Asia, Europe	[36]
*Prunus domestica* *	*Susino* ^a^	Plum	SP	Tree	Asia-Te	[49]
*Prunus persica* *	*Pesco* ^a^	Peach	SP	Tree	Asia-Te	[49]
Pteridophyta	*Felci* ^a^	Ferns	FD	Herbaceous	Africa, Asia, Australasia, Europe, North America, Pacific, South America	[36]
*Pyrus communis* *	*Pero* ^a^	Pear	SP	Tree	Asia-Te, Europe	[49]
*Rhododendron ferrugineum*	*Rosa delle Alpi*	Alpine rose	SP	Shrub	Europe	[36]
*Rhododendron* spp. **	*Azalea/Rododendro* ^a^	Rhododendron	SP	Tree/Shrub	Asia, Australasia, Europe, North America	[36,49]
*Ricinus communis* *	*Ricino* ^a^	Castor bean	FD	Shrub/Herbaceous	Africa	[49]
*Rosa* spp. **	*Rosa rampicante* ^a^	Climbing rose	VP	Climber	Africa, Asia, Europe, North America	[36,49]
*Rosa* spp. **	*Rosa cespugliosa* ^a^	Rose	FD	Shrub	Africa, Asia, Europe, North America	[36,49]
*Salix babylonica* *	*Salice piangente* ^a^	Weeping willow	SP	Tree	Asia-Te	[49]
*Salvia splendens cv. ‘Boule de Feu’* *	*Salvia rossa cv. ‘Boule de Feu’*	Scarlet sage	FD	Herbaceous	South America	[49]
*Salvia* spp. **	*Salvia* ^a^	Sage	FD	Herbaceous	Africa, Asia, Europe, North America, South America	[36,49]
*Spiraea* spp. *	*Spirea*	Spirea	SP	Shrub	Asia-Te, Europe, North America	[49]
*Saxifraga* spp.	*Sassifraghe* ^a^	Saxifrage	FD	Herbaceous	Asia-Te, Europe, North America	[36]
*Tradescantia* spp.	*Tradescantia* ^a^	Spiderwort	HB	Herbaceous	North America, South America	[36]
*Viburnum lantana* *	*Lantana*	Wayfaring tree	FD	Herbaceous	Africa, Asia-Te, Europe	[49]
*Viburnum opulus* *	*Palla di neve*	Guelder rose	SP	Shrub	Africa, Asia-Te, Europe	[49]
*Wisteria sinensis* *	*Glicine* ^a^	Chinese wisteria	VP	Climber	Asia-Te	[49]
*Wisteria* spp.	*Glicine* ^a^	Wisteria	VP	Climber	Asia-Te, North America	[36]
*Yucca* spp. *	*Yucca* ^a^	Yucca	SP	Tree/Shrub	North America	[49]
*Zinnia* spp.	*Zinnie* ^a^	Zinnia	FD	Herbaceous	North America, South America	[36]

No symbols = Taxa employed by stationmasters; * = Taxa recommended by organizers; ** = Taxa recommended and employed; ^a^ = Italian common names reported as in the historical sources; ^b^ = Species deduced from historical sources.

## Data Availability

No new data were created or analyzed in this study.

## Supplementary Materials

The following supporting information can be downloaded at: https://www.mdpi.com/article/10.3390/plants15152282/s1, Table S1: Bibliographic and archival metadata of the materials recovered and related to the Flowery Stations Contest (FSC), held between 1911 and 1914.; Table S2: Data retrieved for documents for the analytical assessment of Flowery Stations Contest (FSC) impact across the 1900s to today. For each station, the FSC year of participation, FSC ranking by year, current name, operational condition (active or closed), region in which it is located, presence or absence of a garden, apparent maintenance condition (“managed” or “neglected”), and any additional notes (e.g., abandonment, damage, reconstruction, station or line closure) were reported. Railway stations were classified according to the historical Italian State Railway (FS/RFI) classification system adopted until 2018 [Gold (main structure), Silver (medium structure), and Bronze (minor structure) categories], as it better reflects the historical and infrastructural relevance of stations, whereas the current classification system is mainly based on operational and service-management criteria.; Figure S1: The original photographs of the Ripafratta railway station preserved in the archive of the *Associazione Salviamo La Rocca di Ripafratta*, together with the aerial photographs from 1954 and 1965 of the station obtained from GeoScopio; Supplementary Material S2: File .kmz of the 90 railway stations investigated in analytical assessment of the impact of the Flowery Station Contest (FSC) over the century.

## Abbreviations

The following abbreviations are used in this manuscript:

FS	*Ferrovie dello Stato Italiane* (Italian State Railways)
FSC	Flowery Stations Contest
IFAC	Italian Federation of Agricultural Consortiums
ITC	Italian Touring Club
RFI	*Rete Ferroviaria Italiana* (Italian Railway Network)
