# Supplementary material for: The Flowery Stations Contest in Early Twentieth-Century Italy: Railway Gardens and the Case Study of Ripafratta (Tuscany)
Source: Plants (Basel). 2026 Jul 25;15(15):2282. doi: 10.3390/plants15152282 (PMC13468185; doi:10.3390/plants15152282)
Supplement: Supplementary file 1 [file plants-15-02282-s001.zip › plants-4377806-supplementary.pdf]

Supplementary Table S1. Bibliographic and archival metadata of the materials recovered and related to the Flowery Stations Contest (FSC), held between 1911 and 1914. FS= *Ferrovie dello Stato Italiane* (Italian State Railways).

Archive or repository	Document	Source type	Quantity of materials recovered	Reference
Central National Library of Florence	Award list and jury report 1914	Booklet	1	[1]
Digitouring.it	Photographs	Image	16	https://www.digitouring.it/it
Emilia Romagna Region website	Map of the railway lines of the 1911 contest	Image	1	[2]
Fondazione Massimo and Sonia Cirulli Archive	Certificate template 1913	Image	1	https://www.fondazionecirulli.org/
National Library of Potenza	Award list 1913	Booklet	1	[3]
Private collector market	Certificate 1913 and medals 1911 and 1913	Image	4	Private collector market
Azienda Agricola vivai e piante Guido Naldini private collection	Certificate 1913	Image	1	https://www.naldinipiante.it/lazienda/
Rivista mensile del Touring Club Italiano 1910	Articles and/or editorial notes	Journal compendium	1	https://www.digitouring.it/it/cerca/40265-rivista-mensile-del-touring-club-italiano-1910
Rivista mensile del Touring Club Italiano 1911	Articles and/or editorial notes	Journal compendium	6	https://www.digitouring.it/it/cerca/40266-rivista-mensile-del-touring-club-italiano-1911
Rivista mensile Touring Club Italiano 1912	Articles and/or editorial notes	Journal compendium	6	https://www.digitouring.it/it/cerca/40267-rivista-mensile-del-touring-club-italiano-1912
Rivista mensile Touring Club Italiano 1913	Articles and/or editorial notes	Journal compendium	5	https://www.digitouring.it/it/cerca/40268-rivista-mensile-del-touring-club-italiano-1913
Rivista mensile Touring Club Italiano 1914	Articles and/or editorial notes	Journal compendium	4	https://www.digitouring.it/it/cerca/40269-rivista-mensile-del-touring-club-italiano-1914
Rivista mensile Touring Club Italiano 1915	Articles and/or editorial notes	Journal compendium	5	https://www.digitouring.it/it/cerca/40270-rivista-mensile-del-touring-club-italiano-1915

Touring Club Italiano Archive	Program booklet 1912	Booklet	1	*Touring Club Italiano* Archive – Milan, Itlay
Touring Club Italiano Archive	Advice and suggestions for the participants booklet 1912	Booklet	1	*Touring Club Italiano* Archive – Milan, Italy
Il progresso fotografico	Articles and/or editorial notes	Journal	1	[4]
Bullettino della Regia Società Toscana di Orticoltura	Articles and/or editorial notes	Journal	1	[5]
Architettura Ferroviaria in Italia	Book chapter	Book	1	[6]
Un Treno per Lucca. Ferrovie e Tranvie in Lucchesia, Valdinievole e Garfagnana, Funicolare di Montecatini	Book	Book	1	[7]
Ordine Di Servizio n. 335 Del 29 Novembre 1909. Bolettino Ufficiale delle Ferrovie dello Stato	Service directive of FS	Service directive	1	[8]
Il Ponte di Pisa	Articles and/or editorial notes	Journal	1	[9]
Il Ponte di Pisa	Articles and/or editorial notes	Journal	1	[10]
Fondazione FS Italiane	Photograph	Image	1	[11]
GEOscopio	Satellite images	Image	2	[12]
Associazione Salviamo la Rocca di Ripafratta Archive	Photographs	Image	16	*Associazione Salviamo la Rocca di Ripafratta* Archive – Ripafratta, San Giuliano Terme, Pisa, Italy
La "stazione fiorita" di Ripafratta. Una nuova vita in verde.	Master's thesis	Thesis	1	[13]

Supplementary Table S2. Data retrieved for documents for the analytical assessment of Flowery Stations Contest (FSC) impact across the 1900s to today. For each station, the FSC year of participation, FSC ranking by year, current name, operational condition (active or closed), region in which it is located, presence or absence of a garden, apparent maintenance condition ("managed" or "neglected"), and any additional notes (e.g., abandonment, damage, reconstruction, station or line closure) were reported. Railway stations were classified according to the historical Italian State Railway (FS/RFI) classification system adopted until 2018 [Gold (main structure), Silver (medium structure), and Bronze (minor structure) categories], as it better reflects the historical and infrastructural relevance of stations, whereas the current classification system is mainly based on operational and service-management criteria. --- = no data.

Year	FSC ranking	Historic name	Current name	FS/RFI classification	Operational condition	Italian region	Garden	Apparent maintenance condition	Note	Reference
1911	1	Nervi	Genova Nervi	Silver	Active	Liguria	Yes	No	---	[14]
	2	Vercelli	Vercelli	Silver	Active	Piedmont	No	---	---	
	3	Pistoia	Pistoia	Silver	Active	Tuscany	No	---	---	
	4	Bolzano	Bolzano/Bozen	Gold	Active	Trentino-Alto Adige	No	---	---	
	5	Salbertrand	Salbertrand	Bronze	Active	Piedmont	No	---	---	
	6	Finalmarina	Finale Ligure Marina	Silver	Active	Liguria	No	---	New building	
	7	Belgirate	Belgirate	Bronze	Active	Piedmont	Yes	No	---	
	8	Avigliana	Avigliana	Silver	Active	Piedmont	No	---	---	
	9	Desio	Desio	Silver	Active	Lombardy	Yes	No	---	
	10	Spotorno	Spotorno-Noli	Silver	Active	Liguria	No	---	---	
	11	Capalbio	Capalbio	Bronze	Active	Tuscany	Yes	No	---	
	12	Peschiera	Peschiera del Garda	Silver	Active	Veneto	Yes	No	---	
	13	Bordighera	Bordighera	Silver	Active	Liguria	Yes	Yes	---	
	14	San Martino della Battaglia	San Martino della Battaglia	Bronze	Active	Lombardy	Yes	No	---	
	15	San Remo	Sanremo	Silver	Active	Liguria	No	---	New building	
	16	Varzo	Varzo	Bronze	Active	Piedmont	Yes	No	---	
	17	Castiglione in Teverina	Castiglione in Teverina	Bronze	Closed	Lazio	No	---	---	

	18	Sturla	Genova Sturla	Silver	Active	Liguria	No	---	---	
	19	Pontenure	Pontenure	Bronze	Active	Emilia-Romagna	Yes	No	---	
	20	Albenga	Albenga	Silver	Active	Liguria	No	---	New building	
	21	Meina	Meina	Bronze	Active	Piedmont	Yes	No	---	
	22	Carimate	Carimate	Bronze	Active	Lombardy	Yes	No	---	
	23	Cambiano Santena	Cambiano-Santena	Bronze	Active	Piedmont	Yes	No	---	
	24	Sant Antonino	Sant'Antonino Vaie	Bronze	Active	Piedmont	No	No	---	
	25	Arcola	Arcola	Bronze	Closed	Liguria	Yes	No	Abandoned building	
	26	Romagnano Sesia	Romagnano Sesia	Bronze	Active	Piedmont	No	---	---	
	27	Maccarese	Maccarese-Fregene	Silver	Active	Lazio	No	---	New building	
	28	Pegli	Genova Pegli	Silver	Active	Liguria	Yes	Yes	---	
	29	Frugarolo	Frugarolo-Boscomarengo	Bronze	Active	Piedmont	Yes	No	---	
	30	Corticella	Bologna Corticella	Bronze	Active	Emilia-Romagna	Yes	No	---	
1912	1	Schio	Schio	Silver	Active	Veneto	Yes	Yes	---	[15]
	3	Belluno	Belluno	Silver	Active	Veneto	Yes	Yes	---	
	4	Montagnana	Montagnana	Bronze	Active	Veneto	Yes	No	---	
	5	Ivrea	Ivrea	Silver	Active	Piedmont	Yes	Yes	---	
	7	Desenzana	Desenzano del Garda-Sirmione	Silver	Active	Lombardy	Yes	No	---	
	8	Santa Margherita Ligure	Santa Margherita Ligure-Portofino	Silver	Active	Liguria	Yes	Yes	---	
	9	Santhià	Santhià	Silver	Active	Piedmont	No	No	---	
	10	Oleggio	Oleggio	Bronze	Active	Piedmont	No	---	New building	
	12	Faenza	Faenza	Silver	Active	Emilia-Romagna	No	---	New building	
	13	Condove	Condove-Chiusa San Michele	Bronze	Active	Piedmont	Yes	No	---	

	15	Bolzano Novarese	Bolzano Novarese	Bronze	Active	Piedmont	Yes	No	---	
	18	Busalla	Busalla	Bronze	Active	Liguria	Yes	No	---	
	19	Codogno	Codogno	Silver	Active	Lombardy	No	---	---	
	20	Castelfranco Emilia	Castelfranco Emilia	Silver	Active	Emilia-Romagna	No	---	New building	
	21	Castelmaggiore	Castel Maggiore	Bronze	Active	Emilia-Romagna	No	---	---	
	22	San Giorgio di Piano	San Giorgio di Piano	Bronze	Active	Emilia-Romagna	Yes	No	---	
	23	San Pietro in Casale	San Pietro in Casale	Bronze	Active	Emilia-Romagna	Yes	No	---	
	25	Piadena	Piadena Drizzona	Bronze	Active	Lombardy	Yes	No	---	
	27	Sesto Calende	Sesto Calende	Silver	Active	Lombardy	Yes	No	---	
	28	Arona	Arona	Silver	Active	Piedmont	No	---	---	
	30	Lesa	Lesa	Bronze	Active	Piedmont	Yes	No	---	
	31	Baveno	Baveno	Silver	Active	Piedmont	Yes	No	---	
	33	Cureggio	Cureggio	Bronze	Closed	Piedmont	Yes	No	Abandoned building	
	34	Gazzada	Gazzada-Schianno-Morazzone	Bronze	Active	Lombardy	Yes	No	---	
	35	Montalto Dora	Montalto Dora	Bronze	Active	Piedmont	No	---	---	
	36	Magnano-Artegna	Artegna	Bronze	Active	Friuli-Venezia Giulia	No	---	New building	
	37	Cornuda	Cornuda	Bronze	Active	Veneto	Yes	No	---	
	38	Camogli	Camogli-San Fruttuoso	Silver	Active	Liguria	Yes	Yes	---	
	39	Sant'Elena d'Este	Sant'Elena-Este	Bronze	Active	Veneto	Yes	No	---	
	40	Buttrio	Buttrio	Bronze	Active	Friuli-Venezia Giulia	Yes	No		
	1	Torre del Lago	Torre del Lago Puccini	Silver	Active	Tuscany	Yes	Yes	---	
1913	3	Castelfiorentino	Castelfiorentino	Silver	Active	Tuscany	Yes	No	---	[3]
	4	Viareggio	Viareggio	Gold	Active	Tuscany	No	---	New building	

5	Castiglione del Lago	Castiglione del Lago	Bronze	Active	Umbria	Yes	No	---
6	Porto San Giorgio	Porto San Giorgio-Fermo	Silver	Active	Marche	Yes	No	---
7	Sesto Fiorentino	Sesto Fiorentino	Silver	Active	Tuscany	No	---	---
8	Cansano	Cansano	Bronze	Active	Abruzzo	Yes	No	---
9	Signa	Signa	Silver	Active	Tuscany	No	---	New building
10	Livorno Centrale	Livorno Centrale	Gold	Active	Tuscany	No	---	---
11	Orbetello	Orbetello-Monte Argentario	Silver	Active	Tuscany	No	---	---
13	Saline di Volterra	Volterra Saline-Pomarance	Bronze	Active	Tuscany	Yes	No	---
14	Ripafratta	Ripafratta	Bronze	Active	Tuscany	Yes	No	---
15	Sulmona-Introacqua	Sulmona-Introacqua	Silver	Closed	Abruzzo	Yes	No	---
16	Ponte a Elsa	Ponte a Elsa	Bronze	Active	Tuscany	Yes	No	---
17	Granaiolo	Granaiolo	Bronze	Active	Tuscany	No	---	New building
18	Vetralla	Vetralla	Bronze	Active	Lazio	Yes	Yes	---
19	Barbarano Romano	Barbarano Romano-Vejano	Bronze	Closed	Lazio	No	---	Abandoned building
20	Potenza Picena	Potenza Picena-Montelupone	Bronze	Active	Marche	Yes	No	---
21	Porto Civitanova	Civitanova Marche-Montegranaro	Silver	Active	Marche	No	---	---
22	San Elpidio al Mare	Porto Sant'Elpidio	Silver	Active	Marche	Yes	No	---
23	San Benedetto del Tronto	San Benedetto del Tronto	Silver	Active	Marche	No	---	---
24	Porto d'Ascoli	Porto d'Ascoli	Silver	Active	Marche	No	---	---
25	Vasto	Vasto-San Salvo	Silver	Active	Abruzzo	No	---	New building

26	Gubbio	Gubbio	---	Closed	Umbria	No	---	Damage during World War 2
27	Urbino	Urbino	---	Closed	Marche	No	---	No active railway line
28	Sipicciano	Sipicciano	Bronze	Active	Lazio	No	---	---
29	Montefiascone	Montefiascone	Bronze	Active	Lazio	No	---	---
30	Cori	Cori	Bronze	Closed	Lazio	No	---	Abandoned building
31	San Giovanni Valdarno	San Giovanni Valdarno	Silver	Active	Tuscany	No	---	---
32	Varano	Varano	Bronze	Active	Marche	No	---	New building

Supplementary Figures S1. The original photographs of the Ripafratta railway station preserved in the archive of the *Associazione Salviamo La Rocca di Ripafratta,* together with the aerial photographs from 1954 and 1965 of the station obtained from GeoScopio [12]**.**

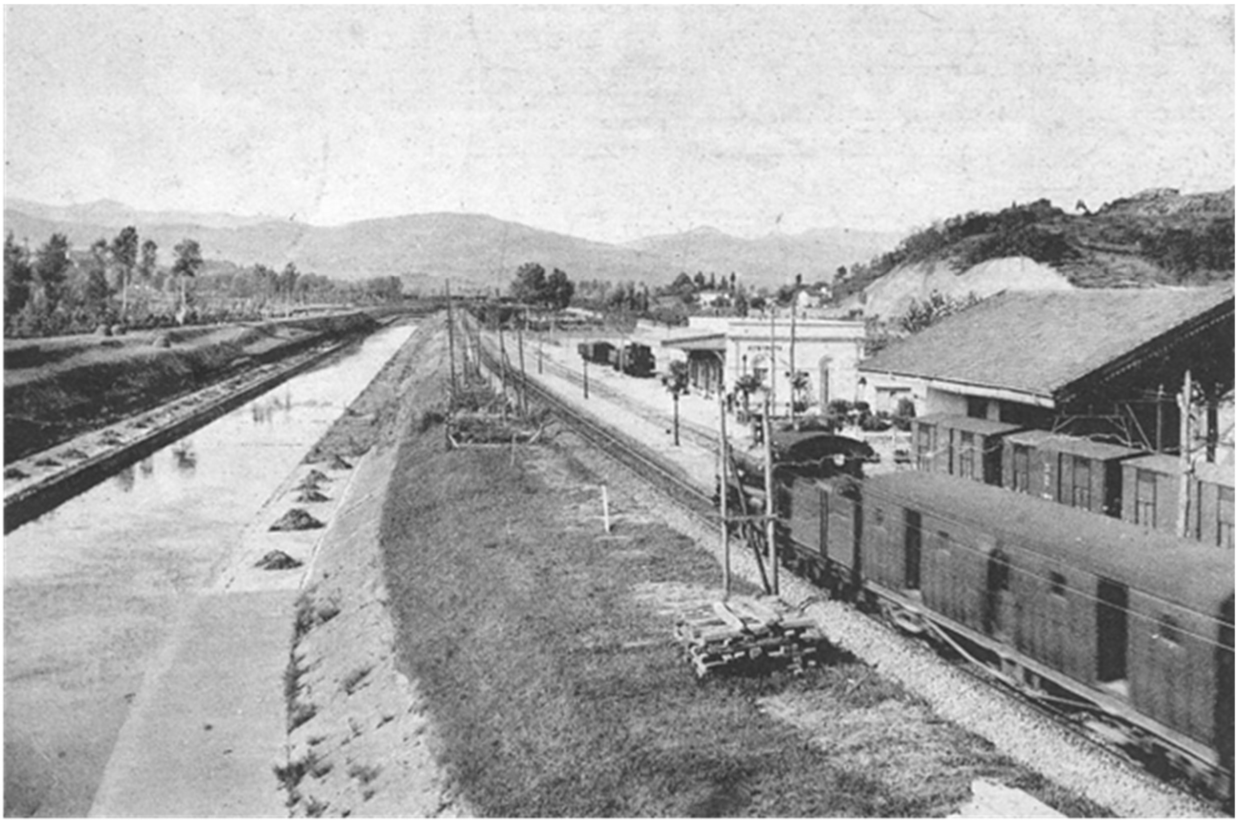

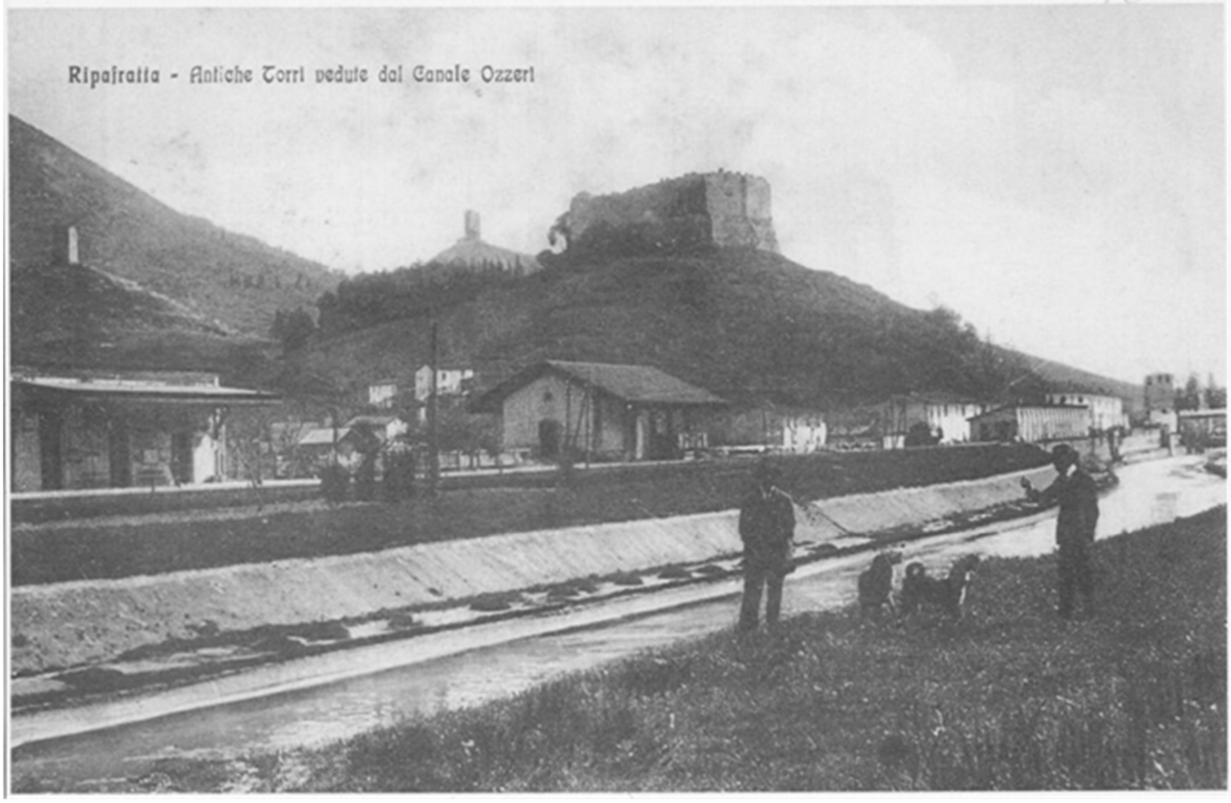

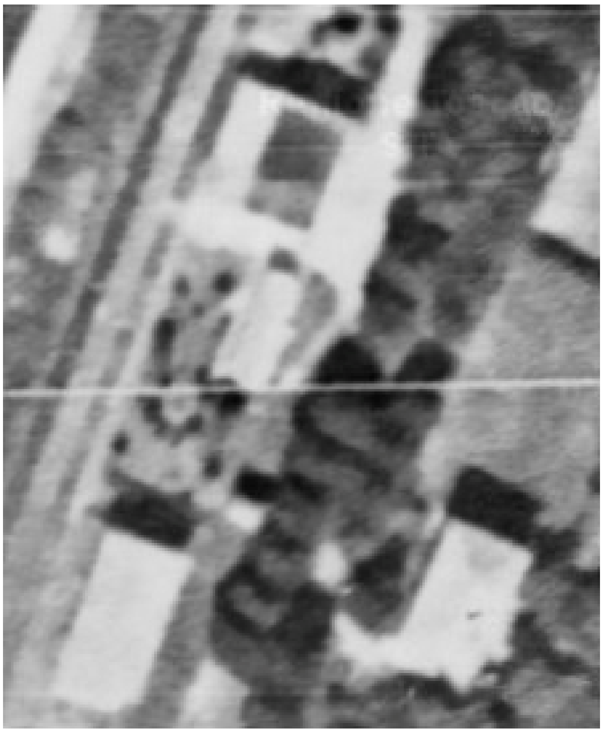

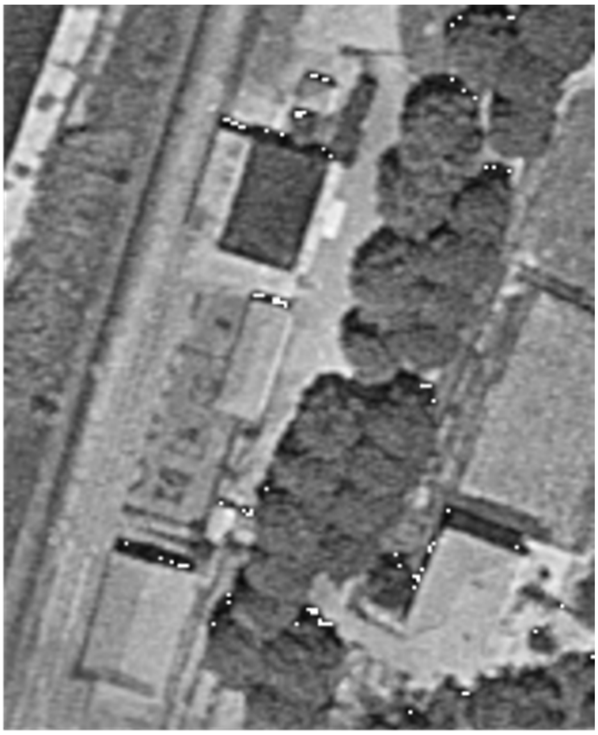

Supplementary references

1. Touring Club Italiano; Federazione Italiana dei Consorzi Agrari; Ferrovie dello Stato Italiane *Concorso Fra Le Stazioni Fiorite 1914 Indetti Dal Touring Club Italiano e Dalla Federazione Italiana Dei Consorzi Agrari Con l'appoggio Delle Ferrovie Dello Stato.*; Touring Club Italiano: Milano, 1915; Vol. 1; pp. 1-23.
2. Morsia, D. In Redazione Arriva La Primavera Con Il Concorso Delle Stazioni Fiorite Available online: https://agricoltura.regione.emilia-romagna.it/agricultura/agricoltura-editoria-del-900/la-voce/biblioteca-piacenza-passerini-landi/concorso-stazioni-fiorite-in-redazione-arriva-la-primavera (accessed on 14 March 2026).
3. Touring Club Italiano; Federazione Italiana dei Consorzi Agrari; Ferrovie dello Stato Italiane *Elenco Di Premiazione. Concorso Delle Stazioni Fiorite 1913 Indetto Dal Touring Club Italiano e Dalla Federazione Italiana Dei Consorzi Agrari Coll'appoggio Ed Il Contributo Delle Ferrovie Dello Stato.*; Touring Club Italiano: Milano, 1914; Vol. 1; pp. 1-10.
4. Anonymous Concorso Delle Stazioni Fiorite. Iniziative Utili. *Il Prog. Fotogr.* **1912**, *1*, 163–164.
5. Anonymous I Concorsi Delle Stazioni Fiorite. Dai Periodici Orticoli. *Bull. Della Regia Soc. Toscana Ortic.* **1912**, 125–126.
6. Brunetti, F. *I concorsi delle stazioni fiorite indetti dal Touring Club Italiano in Architettura ferroviaria in Italia. Novecento*; Ezio Godoli, Lima, A.I., Eds.; Flaccovio: Palermo, 2004; Vol.1; ISBN 978-88-7758-597-4; pp. 1-520.
7. Carboncini, A.B. *Un Treno per Lucca. Ferrovie e Tranvie in Lucchesia, Valdinievole e Garfagnana, Funicolare Di Montecatini*; Calosci: Cortona, 1990; Vol. 1; ISBN 978-88-7785-044-7; pp. 1-270.
8. Ferrovie dello Stato italiane Ordine Di Servizio n. 335 Del 29 Novembre 1909. *Boll. Uff. Delle Ferrov. Dello Stato* **1909**, 791–793.
9. Il Mattatino L'Esposizione Dei Crisantemi Etc. A Piè Del Ponte. *Il Ponte di Pisa* **1905**, *42*, 3. Available online: https://www.internetculturale.it/jmms/iccuviewer/iccu.jsp?id=oai%3Awww.internetculturale.sbn.it%2FTeca%3A20%3ANT0000%3ACFI0361836_160802&mode=all&teca=MagTeca+-+ICCU&fulltext=1 (accessed on 20 April 2026).
10. Il Duchino Teste e Tasti. *Il Ponte di Pisa* **1914**, *12*, 2. Available online: https://www.internetculturale.it/jmms/iccuviewer/iccu.jsp?id=oai%3Awww.internetculturale.sbn.it%2FTeca%3A20%3ANT0000%3ACFI0361836_161239&mode=all&teca=MagTeca+-+ICCU&fulltext=1(accessed on 20 April 2026).
11. Fondazione FS Italiane NEG_17084N_18014V_MSTR_MC062.Tif Available online: https://www.archiviofondazionefs.it/it/search/?text=NEG_17084N_18014V_MSTR_MC062&selectMode=PHOTOS (accessed on 16 April 2026).
12. Tuscany Region GEOscopio Available online: https://www.regione.toscana.it/-/geoscopio (accessed on 16 April 2026).
13. Pedrelli, A. *La "Stazione Fiorita" Di Ripafratta. Una Nuova Vita in Verde*. University of Pisa, Department of Agricultural, Food and Agro-Environmental Sciences, Master's Degree in Urban Green and Landscape Design and Management, Pisa, Italy, 2019.
14. Anonymous I Concorsi Delle Stazioni Fiorite 1911 e 1912. *Riv. Mens. Tour. Club Ital.* **1912**, *1*, 218.
15. Anonymous Concorso Fra Le Stazioni Fiorite. Il Concorso 1912. *Riv. Mens. Tour. Club Ital.* **1913**, *1*, 86.

Acknowledgments: Images and photographs reproduced in this manuscript are included solely for scholarly, educational, and documentary purposes. Their use is intended exclusively to support academic discussion and historical documentation within the context of this study. All copyrights and related rights remain the property of their respective owners. The authors make no claim of ownership over third-party materials and have made every reasonable effort to acknowledge and credit the original sources and rights holders where known.
